# NRF1 is upregulated by docosahexaenoic acid to ameliorate MASH through the inhibition of ER stress

**DOI:** 10.1038/s41419-025-08139-1

**Published:** 2026-01-16

**Authors:** Mengchi Lin, Hongtao Zhang, Shuai Chen, Jie Zhang, Chenxi Tang, Xin Song, Jiaming Zhou, Zixin Xu, Yali Mu, Hang Zeng, Changqing Yang, Chaohui Yu, Chengfu Xu

**Affiliations:** 1 Department of Gastroenterology, Zhejiang Provincial Clinical Research Center for Digestive Diseases, the First Affiliated Hospital, Zhejiang University School of Medicine, Hangzhou, China; 2 Department of Biochemistry, Zhejiang University School of Medicine, Hangzhou, China; 3 Department of Gastroenterology and Hepatology, Tongji Hospital, School of Medicine, Tongji University, Shanghai, China

**Keywords:** Non-alcoholic steatohepatitis, Proteasome, ER-associated degradation, Chronic inflammation, Mechanisms of disease

## Abstract

Despite the high prevalence of metabolic dysfunction-associated steatohepatitis (MASH), the number of effective therapeutic targets is limited due to a vague understanding of its intricate pathogenesis. In this study, we reported that the expression of nuclear factor erythroid-derived 2-related factor 1 (NRF1), an endoplasmic reticulum (ER) membrane-bound transcription factor that governs the expression of proteasome subunit genes, was significantly reduced in liver tissues from MAFLD patients and from mice fed a high-fat diet (HFD) for 20 weeks. Liver-specific overexpression of NRF1 in mice markedly ameliorated HFD-driven hepatic steatosis, liver injury and inflammation. Elevated NRF1 expression restored the function of the proteasome, facilitating the degradation of unfolded and nonfunctioning proteins, thereby mitigating ER stress and reducing oxidative stress. Moreover, docosahexaenoic acid (DHA) was found to increase NRF1 expression, contributing to the amelioration of MASH. Mechanistically, DHA inhibited the ubiquitination of NRF1 via the cytoplasmic E3 ligases FBW7 and HRD1 at the ER membrane, thereby preventing its degradation. Liver-specific knockdown of NRF1 abrogated the protective effect of DHA on HFD-driven MASH in mice. Together, our findings underscore the pivotal role of NRF1 in the DHA-mediated amelioration of MASH and suggest that NRF1 is a potential therapeutic target for MASH management.

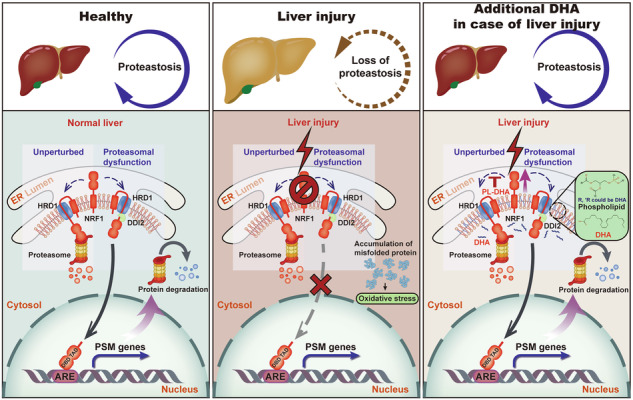

## Introduction

With changes in global dietary patterns and worldwide increases in obesity prevalence, the incidence of metabolic dysfunction-associated fatty liver disease (MAFLD) is increasing annually [1]. While most patients with MAFLD remain in the steatosis stage, 20% of them develop metabolic dysfunction-associated steatohepatitis (MASH), which is characterized by liver injury, inflammation and fibrosis [2]. Despite the prevalence of MASH, effective therapeutic options are limited due to a vague understanding of its complex pathogenesis. Multiple factors, including genetic predisposition, endoplasmic reticulum (ER) stress, oxidative stress and apoptosis, contribute to severe liver injury in MASH patients [3, 4]. As the basic feature of MASH, liver injury initiates the release of molecules termed damage-associated molecular patterns (DAMPs), further leading to inflammation and fibrosis [5]. Thus, targeting metabolic pathways to alleviate liver injury is an attractive paradigm for therapeutic intervention.

The ubiquitin‒proteasome system (UPS) is indispensable for clearing damaged and nonfunctioning proteins, preserving the balance of the cellular amino acid pool and maintaining cellular proteostasis [6, 7]. Accumulating evidence suggested that dysregulated proteostasis led to the aggravation of liver damage [8]. In this study, we focused on the hepatotoxicity of proteasome inhibitors, such as ixazomib and bortezomib, in clinical practice. Cases of clinically apparent liver injury with elevated serum aminotransferase levels have been frequently reported in patients receiving proteasome inhibitors [9, 10]. However, the role of the proteasome in MASH-related liver injury and its specific regulatory mechanisms remain poorly understood. Nuclear factor erythroid-derived 2-related factor 1 (NRF1; also known as NFE2L1), an ER-localized transcription factor of the cap “n” collar (CNC) basic leucine zipper family, was reported as the main regulator responsible for the transcription of proteasome subunits in mammals [11, 12]. Therefore, we aimed to explore the potential role of NRF1-mediated regulation of proteasome transcription in the progression of MASH.

NRF1 is often considered a negative feedback regulator of proteasome abundance to prevent proteotoxicity; however, the natural regulatory mechanism of NRF1 in hepatocytes is poorly understood. Given the potential for NRF1 to serve as a sensor responding to the lipid component in the bilayer lipid membrane [13] and the potential regulatory role of docosahexaenoic acid (DHA, C22:6, n-3) in proteasome activity in sarcopenia [14, 15], we considered DHA a natural regulator of NRF1. DHA is an essential ω-3 polyunsaturated fatty acid (PUFA) known for its strong anti-inflammatory properties, as it inhibits leukocyte chemotaxis and blocks the secretion of proinflammatory cytokines [16]. Previous reports have demonstrated the beneficial role of DHA in diverse liver diseases, including MAFLD and cirrhosis [17, 18]. We hypothesized that DHA may ameliorate MASH by affecting NRF1 expression.

In this study, we showed that NRF1 expression was lower in patients with MAFLD than in healthy individuals. We identified NRF1 as a fundamental regulator that alleviated MASH-related liver injury by promoting proteasome-mediated protein degradation and further inhibiting ER stress. In addition, NRF1 expression was upregulated by DHA, mediating its protective effect on MASH. Mechanistic work indicated that DHA inhibited interactions between NRF1 and its E3 ligases at the ER membrane, thereby preventing its ubiquitination and subsequent degradation through the UPS, resulting in the accumulation of full-length NRF1.

## Materials and methods

Additional methods are described in the Supplemental Methods.

### Patient samples

Human liver samples from healthy individuals (*n* = 10) and MAFLD patients (*n* = 20) were collected from the First Affiliated Hospital, Zhejiang University School of Medicine. All the samples were subjected to immunohistochemical staining for NRF1, which was analysed using ImageJ software. Briefly, the percentage of NRF1-positive areas in 10 images per sample (scale bar = 50 µm) was calculated. The study protocol conformed to the ethical guidelines of the 2013 Declaration of Helsinki, abided by the 2018 Declaration of Istanbul and was approved by the Ethics Committee of the First Affiliated Hospital, Zhejiang University School of Medicine. Informed consent was obtained from all participating patients included. This study was a retrospective analysis in which residual liver samples from patients with a clinical diagnosis of MAFLD, including simple steatosis and MASH, were used.

### Animal models

Six-week-old male C57BL/6 mice were purchased from Hangzhou Medical College (Hangzhou, China). All the mice were housed in a temperature-controlled environment (23 ± 2 °C) under a 12 h light/dark cycle and provided a standard diet. All the mice had free access to water and food during the experiments. Hepatocyte-specific NRF1-knockdown or NRF1-overexpressing C57BL/6 mice were generated via an intravenous tail injection of AAV-8 obtained from Vigene Biosciences Co. Ltd. (Shandong, China). The primer sequence used to generate AAV8-shNRF1 was 5’-GGAAATGCAGGCTATGGAAGTTTCAAGAGAACTTCCAT

AGCCTGCATTTCCTTTTTT-3’. The nonsense sequence was 5’-TTCTCCGAACGTGTCACGTTTCAAGAGAACGTGACACGTTCGGAGAATTTTTT-3’. Four weeks after viral injection, the mice were randomly divided into groups for subsequent modelling experiments.

The mice were fed a high-fat diet (HFD) containing 60 kcal% fat (PD6001) with or without 4% DHA (PD22092701) for 20 weeks to model MASH. To establish another MASH model, the mice were acclimated to a standard chow diet (SCD) for 1 week and then randomly divided into three groups: (a) the control group, in which the mice continued with the SCD; (b) the CCl_4_ group, in which the mice continued with the SCD and were intraperitoneally injected with a 5 μL/g CCl_4_ working solution (1 mL of CCl_4_ dissolved in 4 mL of corn oil) twice weekly; and (c) the WD+CCl_4_ group, in which the mice were fed a Western diet (WD) (21.1% fat, 41% sucrose, and 1.25% cholesterol) and were also intraperitoneally injected with the CCl_4_ working solution at the same dose. All groups of mice were fed their respective diets for 3 months. All the rodent diets were purchased from Changzhou SYSE Biotec Co., Ltd. (the DHA feed formula is shown in Table S1). Body weights and food intake were monitored every week during the treatment phase. Liver tissues, adipose tissues and blood samples were harvested at the end of the experiment. The hepatic pathologies were assessed via haematoxylin‒eosin (H&E) staining and Masson’s trichrome staining, which were conducted by Biossci Biotechnology Co. Ltd. (Wuhan, China). Serum biochemical indices, including alanine transaminase (ALT) and aspartate transaminase (AST) levels, were analysed with an alanine aminotransferase assay kit (C009-2-1; Nanjing Jiancheng Bioengineering Institute) and an aspartate aminotransferase assay kit (C010-2-1; Nanjing Jiancheng Bioengineering Institute), respectively. All mouse studies were approved by the Animal Care and Use Committee of the First Affiliated Hospital, Zhejiang University School of Medicine.

### Bioinformatics analysis

Publicly available raw transcriptomic data were retrieved from the Gene Expression Omnibus (GEO) database (http://www.ncbi.nlm.nih.gov/geo/). NRF1 expression was analysed in liver tissues from patients with MAFLD or MASH (GEO accession number: GSE126848). The hepatic expression profiles of MASH patients (*n* = 5) and MAFL patients (*n* = 5) were obtained from the GSE105127 dataset. The pathway enrichment analysis was performed via DAVID (https://david.ncifcrf.gov/summary.jsp).

### Cell culture

The human hepatic cell lines HepG2 and Huh7 were cultured in Dulbecco’s modified Eagle’s medium (DMEM) (Gibco) supplemented with 10% foetal bovine serum (FBS). The mouse hepatic cell line AML12 was cultured in DMEM/Nutrient Mixture F-12 (1:1) supplemented with 10% FBS. All the cell lines were authenticated by STR profiling and tested with no mycoplasma contamination. Cells were cultured in a 37 °C constant temperature incubator with 5% CO_2_. After reaching 90% confluence, the cells were washed with phosphate-buffered saline (PBS) followed by trypsinization. Lipofectamine 3000 was used for plasmid transfection according to the manufacturer’s instructions. The NRF1-KO cell line was generated using the CRISPR/Cas9 system and selected with puromycin. Monoclonal cell lines were created via the limiting dilution method.

Cells at a 70–80% density were treated with 1 mM free fatty acids (FFAs) at a ratio of 2:1 (v/v) oleic acid (OA) and palmitic acid (PA) in serum-free medium for 24 h to model MASH. Control cells were provided with bovine serum albumin (BSA) at the same dose. Next, the cells were treated with 10 μM MG132 (HY-13259, MCE) for 6 h to inhibit proteasome activity or 2 μg/mL tunicamycin (HY-A0098, MCE) for 24 h to induce ER stress.

### Statistics

All analysed results are presented as the means ± standard errors of the means (SEMs). Statistical analyses were performed with GraphPad Prism 9.0. For comparisons between two groups, an unpaired Student’s *t* test was used for the statistical analysis. For comparisons among more than two groups with a single variable, one-way ANOVA followed by Dunnett’s multiple pairwise comparison test was applied for evaluation. For comparisons among groups with more than one variable, two-way ANOVA followed by Tukey’s multiple pairwise comparison test was used for the analysis. Spearman’s correlation analysis was performed using SPSS Statistics 22.0. A *P* value less than 0.05 was considered statistically significant, and statistical significance is indicated by asterisks: ns, not significant; *P < 0.05, **P < 0.01, and ***P < 0.001.

## Results

### Hepatic NRF1 expression was reduced in MASH patients and models

**Fig. 1:**
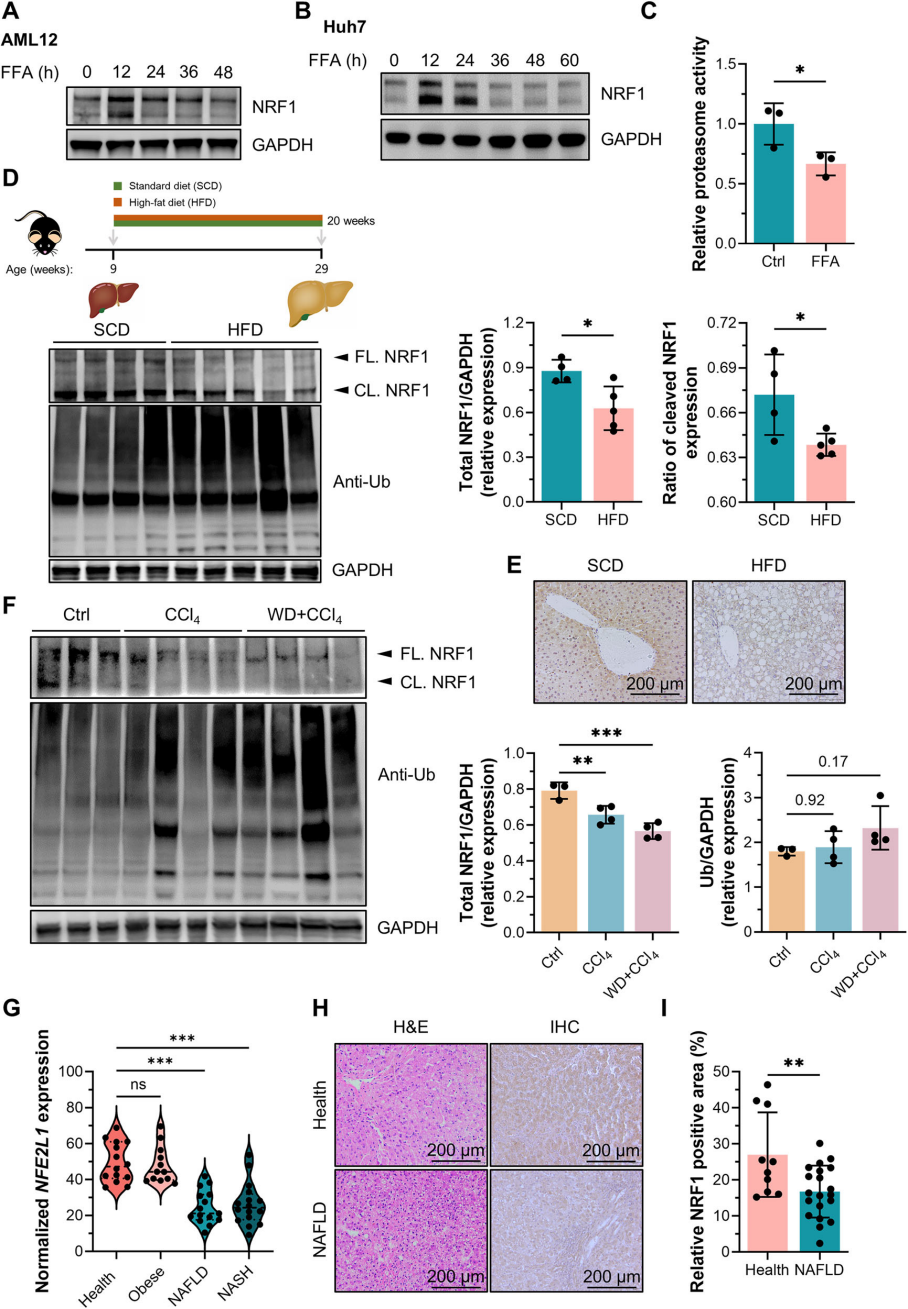
Decreased hepatic NRF1 expression and reduced proteasomal activity in MASH patients and models. **A**, **B** Western blot analysis of NRF1 expression in AML12 cells (**A**) and Huh7 cells (**B**) treated with 1 mM FFAs for different durations. **C** Proteasomal activity in FFA-treated HepG2 cells detected by the fluorogenic substrate suc-LLVY-amc (*n* = 3 for each group). **D** Western blot analysis of NRF1 and ubiquitin expression in livers of mice fed an HFD for 20 weeks (SCD, *n* = 4; HFD, *n* = 5). **E** Immunohistochemical staining for NRF1 in livers of mice fed an SCD or HFD for 20 weeks. **F** Western blot analysis of NRF1 expression in livers of mice treated with CCl_4_ with or without a WD (Ctrl, *n* = 3; CCl_4_, *n* = 4; WD+CCl_4_, *n* = 4). **G** The gene expression of NRF1 in liver tissues from healthy individuals (*n* = 14), obese individuals (*n* = 12), patients with MAFLD (*n* = 15) and patients with MASH (*n* = 16). The analysed data were collected from the GEO dataset GSE126848. **H** Liver sections from healthy people (*n* = 10) and patients with MAFLD (*n* = 20) were analysed via H&E staining and immunohistochemistry for NRF1. Representative images are shown; scale bar = 200 µm. **I** Percentage of NRF1-positive areas per field of view in samples from healthy controls and MASH patients. The data were plotted as the means ± SEMs. Two-tailed Student’s *t* test in (**C**, **D** and **I**) and one-way ANOVA in (**F**, **G**) were used for the statistical analyses. ns: not significant, * *P* < 0.05, ** *P* < 0.01, and *** *P* < 0.001. FL full-length, CL cleaved.

We extracted data from the GEO database to explore the potential connection between MASH progression and proteasome function and observed differential expression of genes involved in the UPS between MASH patients and MAFL patients (GSE105127) (Fig. S1A). We also found that NRF1 expression was upregulated in AML12 and Huh7 cells when treated with FFAs for 12 h, but prolonged treatment with FFAs led to a significant decrease in NRF1 expression (Fig. 1A, B). Furthermore, proteasome activity was inhibited in the cellular model of MASH (Fig. 1C). We also examined NRF1 expression profiles in diet-induced MASH model in mice and found that hepatic NRF1 expression was markedly downregulated, whereas ubiquitin expression, reflecting the degradative ability of the proteasome, was increased in liver tissues from mice fed an HFD (60% caloric intake provided by saturated fat) compared with those from mice fed an SCD for 20 weeks (Fig. 1D).

The IHC analysis not only confirmed the downregulation of NRF1 expression in liver tissues from HFD-fed mice but also demonstrated that NRF1 was predominantly expressed around the central vein rather than the periportal area (Fig. 1E). Additionally, an analysis of liver zonation profiles obtained from the GEO database (GSE84498) revealed a gradual decrease in NRF1 expression from the central vein towards the periportal area (Fig. S1B, C). This pattern aligned with the trend of the variation in the proteasome pathway (Fig. S1D). Additionally, in a chronic liver fibrosis model (CCl_4_ treatment with or without a Western diet), hepatic NRF1 expression was decreased while ubiquitin expression was increased (Fig. 1F). Moreover, we analysed the NRF1 expression profile of patients with MAFLD or MASH using data from the GEO database (GSE126848) and found that NRF1 expression remained unchanged in obese individuals compared with healthy controls but was significantly decreased in patients with MAFLD or MASH (Fig. 1G). We then collected liver tissues from patients with pathologically confirmed MAFLD and found that NRF1 expression was also significantly reduced (Fig. 1H, I).

### Liver-specific overexpression of NRF1 ameliorated MASH

**Fig. 2:**
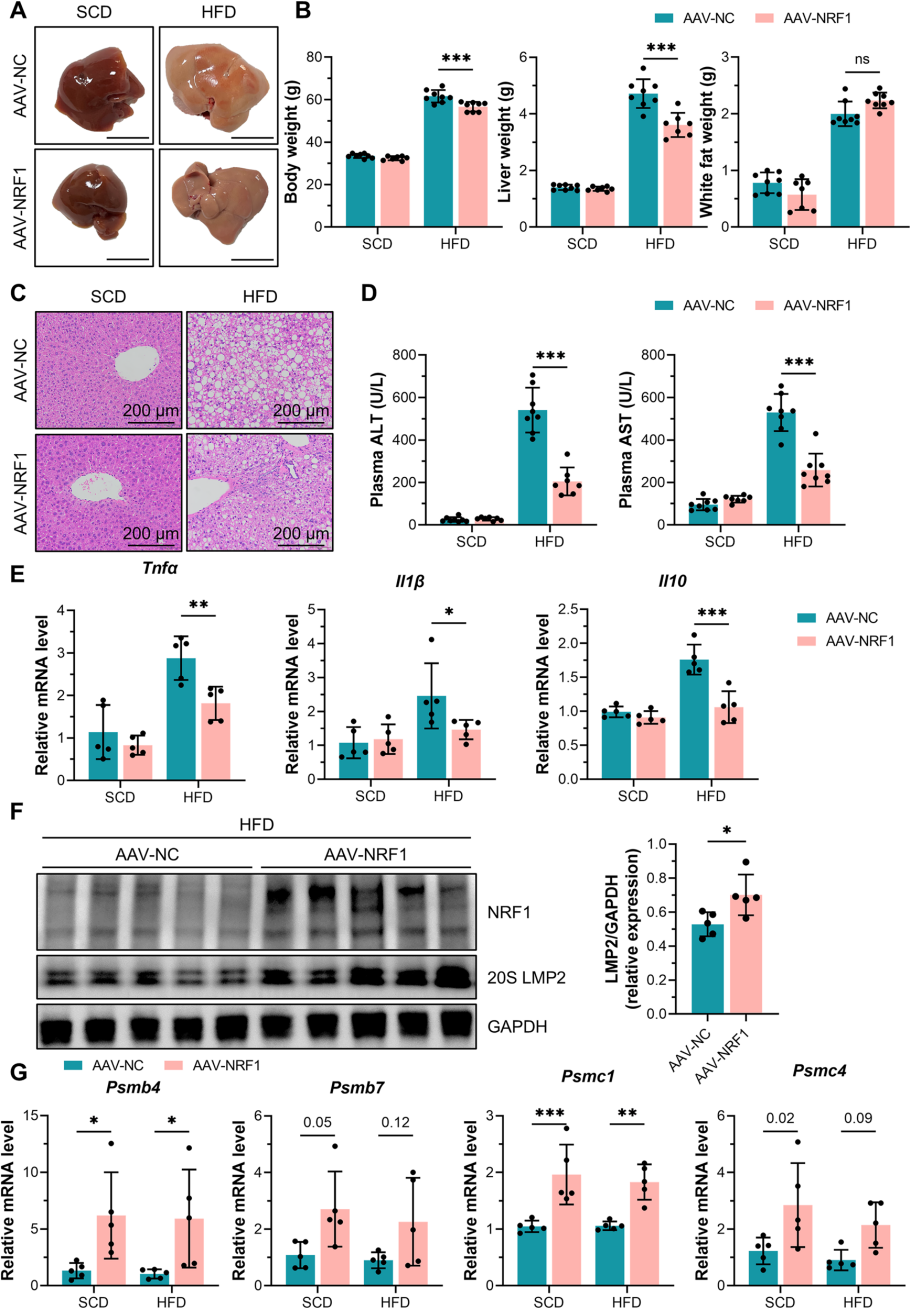
Liver-specific NRF1 overexpression ameliorated MASH in mice. **A** Gross observation of livers from WT and NRF1-overexpressing mice fed the SCD or HFD for 20 weeks. Scale bar, 10 mm. **B** Body weight, liver weight and white fat weight of WT and NRF1-overexpressing mice fed the SCD or HFD (*n* = 8 mice/group). **C** H&E staining of liver tissues from WT and NRF1-overexpressing mice fed the SCD or HFD. **D** Aminotransferase levels in the serum of WT and NRF1-overexpressing mice fed the SCD or HFD (*n* = 8 mice/group). **E** The gene expression of inflammatory factors (*Tnfα*, *Il1β* and *Il10*) in livers of the abovementioned mice (*n* = 5 for each group). **F** Western blot analysis of NRF1 and LMP2 expression in livers of the abovementioned mice (*n* = 5 for each group). **G** The gene expression of proteasome subunits (*Psmb4*, *Psmb7*, *Psmc1* and *Psmc4*) in livers of the abovementioned mice (*n* = 5 for each group). The data were plotted as the means ± SEMs. Two-tailed Student’s *t* test in (**F**) and two-way ANOVA in (**B**, **D**, **E**, **G**) were used for the statistical analyses. ns: not significant, * *P* < 0.05, and *** *P* < 0.001.

We generated mice with AAV8-driven hepatic overexpression of NRF1 and then fed them an HFD for 20 weeks to determine the role of NRF1 in MASH progression. Liver-specific overexpression of NRF1 ameliorated liver discolouration and the hypertrophic volume induced by HFD feeding, as observed through a gross examination of the liver (Fig. 2A). In addition, we found that NRF1 overexpression significantly reduced both the body weight and liver weight, whereas the white fat weight remained unaffected, suggesting a specific protective effect of NRF1 on the liver rather than on general metabolism (Fig. 2B). H&E staining of hepatic tissues revealed that NRF1 overexpression alleviated hepatic steatosis, hepatocyte ballooning and inflammatory cell infiltration (Fig. 2C), which are typical characteristics of MASH [19]. Furthermore, liver-specific NRF1 overexpression reduced the elevated serum aminotransferase levels induced by HFD feeding (Fig. 2D). Inflammation was also alleviated, as indicated by the decreased transcriptional levels of inflammatory factors (*Tnfα*, *Il1β* and *Il10*) (Fig. 2E). Considering that NRF1 is the major transcription factor regulating proteasome subunits, we aimed to explore the NRF1-mediated regulation of proteasomal abundance in MASH models. Both the transcriptional and protein levels of proteasome subunits were increased in NRF1-overexpressing mice compared with WT controls (Fig. 2F, G), indicating that the increased proteasomal abundance led to the degradation of nonfunctioning proteins.

### DHA upregulated NRF1 expression and facilitated its nuclear translocation

**Fig. 3:**
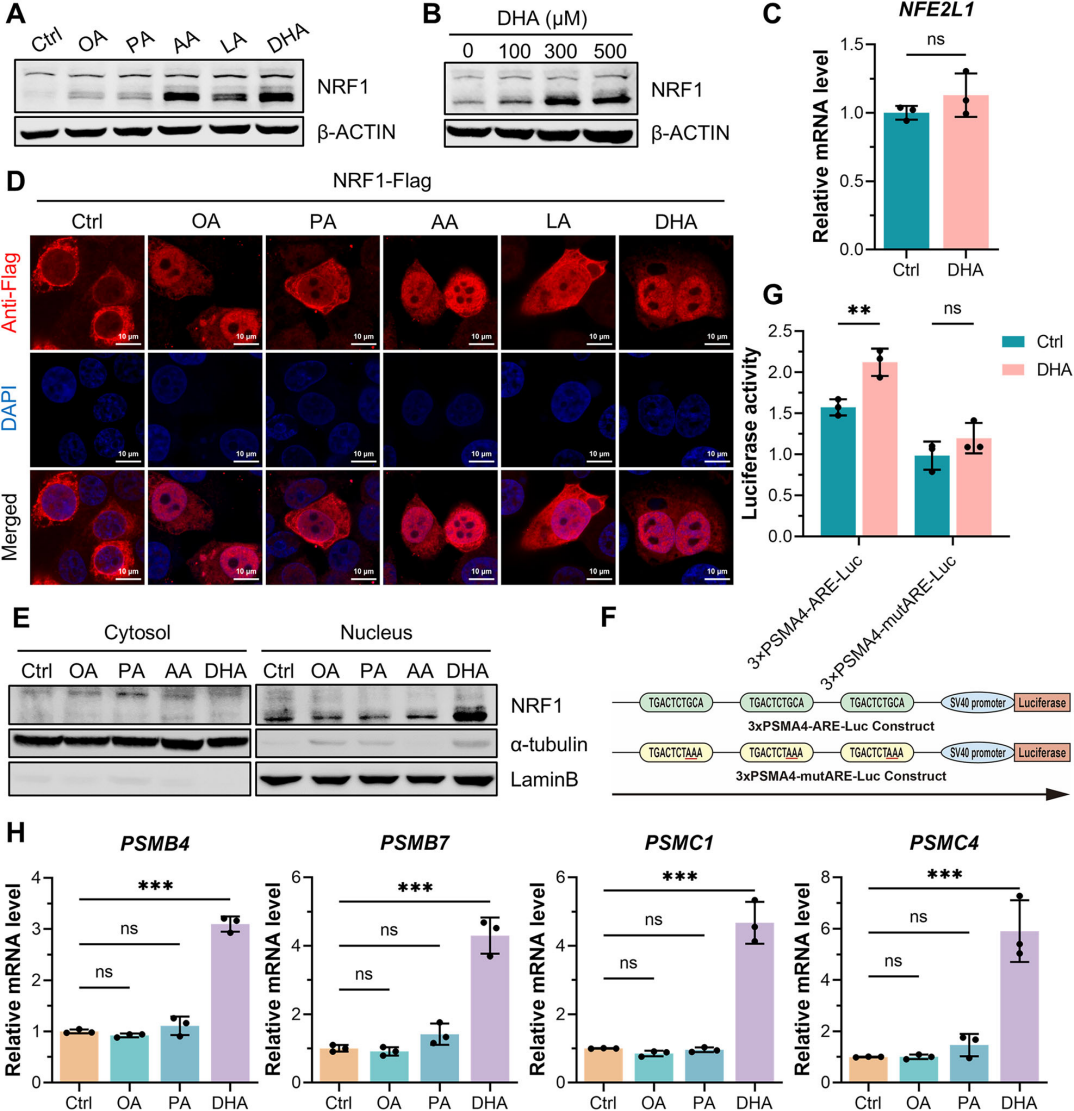
DHA upregulated NRF1 expression and promoted its nuclear translocation. **A** Western blot analysis of NRF1 expression in HepG2 cells treated with various fatty acids (0.1 mM) for 12 h. **B** Western blot analysis of NRF1 expression in HepG2 cells stimulated with increasing concentrations of DHA. **C** The gene expression of NRF1 in HepG2 cells following DHA stimulation (*n* = 3 for each group). **D** Immunofluorescence staining of NRF1-Flag-overexpressing HepG2 cells stimulated with various fatty acids. **E** NRF1 expression in the nucleus and cytoplasm was separately analysed through nuclear and cytoplasmic separation assays after stimulation with various fatty acids. **F** Schematic diagram of the construction of the 3×PSMA4-ARE-Luc plasmid and the 3×PSMA4-mutARE-Luc plasmid used in the luciferase reporter gene assay. **G** Luciferase reporter gene assay in HepG2 cells overexpressing NRF1-Flag upon DHA stimulation (*n* = 3 for each group). **H** The gene expression of proteasome subunits (*PSMB4*, *PSMB7*, *PSMC1,* and *PSMC4*) in HepG2 cells stimulated with 0.1 mM OA, PA or DHA for 12 h (*n* = 3 for each group). The data were plotted as the means ± SEMs. Two-tailed Student’s *t* test in (**C**, two-way ANOVA in (**G**) and one-way ANOVA in (**H**) were used for the statistical analyses. ns: not significant, ** *P* < 0.01, and *** *P* < 0.001.

To determine the regulatory mechanism of NRF1 in MASH, we detected NRF1 expression in hepatocytes stimulated with different fatty acids. In HepG2 and AML12 cells, stimulation with various fatty acids individually upregulated NRF1 expression. Specifically, palmitic acid, a saturated fatty acid (SFA), and oleic acid, a monounsaturated fatty acid (MUFA), slightly increased NRF1 expression, whereas arachidonic acid (AA), linolenic acid (LA) and DHA, which are polyunsaturated fatty acids (PUFAs), dramatically increased NRF1 expression (Figs. 3A and S2B). Moreover, NRF1 expression increased in a concentration-dependent manner in response to DHA (Fig. 3B). Notably, DHA primarily increased the expression of the cleaved form of NRF1 but not the full-length form. However, no distinct difference in the transcriptional level of NRF1 was observed between DHA-treated hepatic cells and unstimulated hepatic cells (Fig. 3C), suggesting that DHA contributed to increased NRF1 expression mainly through the inhibition of degradation rather than the promotion of transcription.

Considering that the cleaved form of NRF1 could be transferred into the nucleus and function as a transcription factor [11, 12], we sought to assess whether DHA affected the nuclear entry of NRF1. As indicated by immunofluorescence of HepG2 cells, the majority of NRF1 was located in the cytoplasm without any stimulation, and unsaturated fatty acids, including MUFAs and PUFAs, promoted its nuclear entry, whereas stimulation with PA did not significantly increase the nuclear translocation of NRF1 (Fig. 3D). Additionally, a nuclear and cytosolic fractionation assay confirmed that, compared with other fatty acids, DHA promoted the nuclear entry of NRF1 more prominently (Fig. 3E). A luciferase reporter assay also revealed that DHA indeed facilitated the transcriptional function of NRF1; however, mutation of the antioxidant response element (ARE), a cis-acting transcriptional regulatory element which NRF1 could bind to, abolished the promoting effect of DHA on NRF1 function (Fig. 3F, G). Predictably, the mRNA level of proteasome subunits was also increased by DHA (Fig. 3H). Taken together, our results suggested that, compared with other fatty acids, DHA upregulated NRF1 and promoted its nuclear translocation more prominently.

### DHA suppressed the ubiquitination and degradation of NRF1

**Fig. 4:**
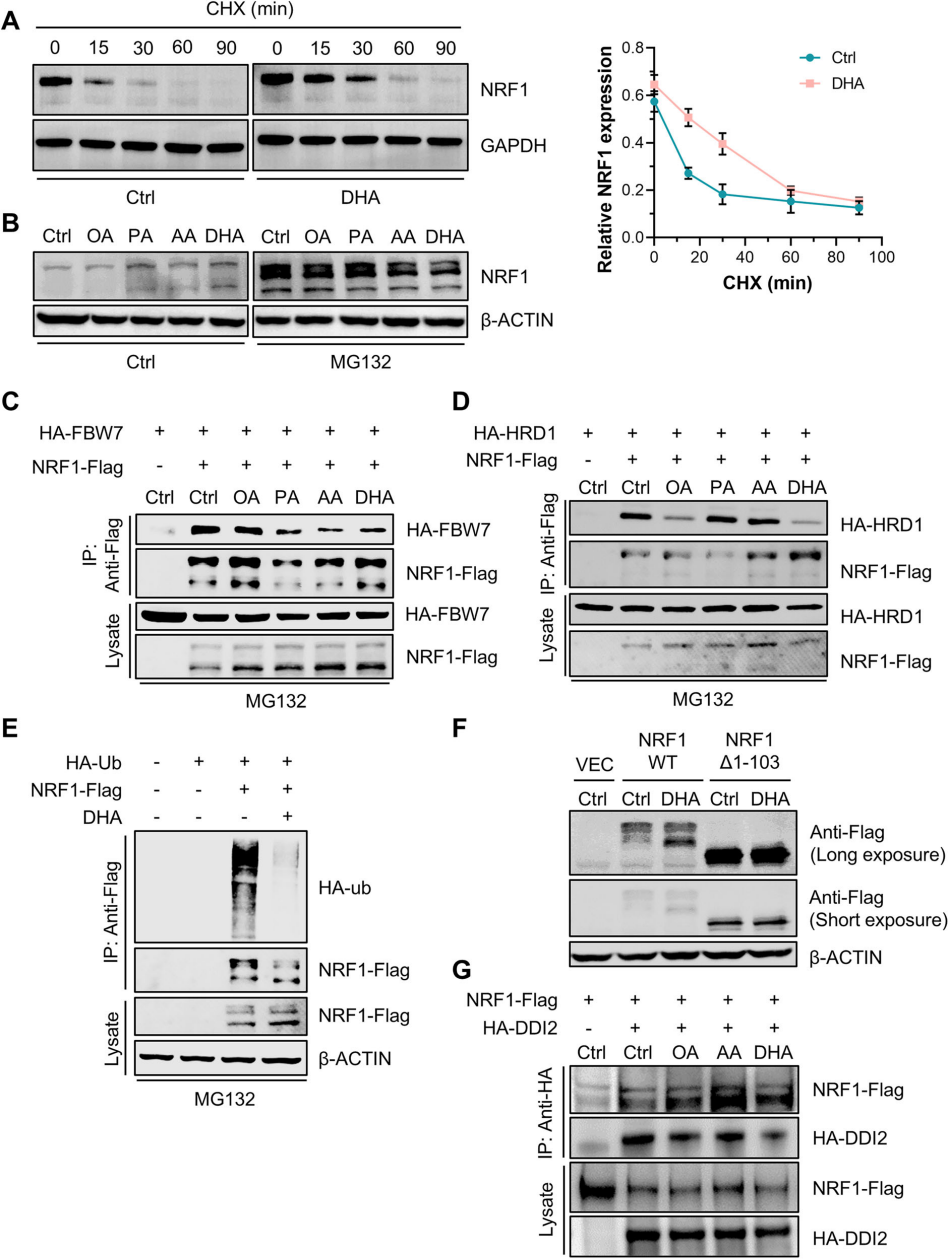
DHA inhibited the ubiquitination and degradation of NRF1 at the ER membrane. **A** The half-life of NRF1 in HepG2 cells after DHA stimulation, as indicated by 10 μg/mL CHX treatment for various durations (*n* = 3 for each group). **B** Synthesis of NRF1 in HepG2 cells in response to stimulation with various fatty acids, as indicated by 10 μM MG132 treatment for 6 h. **C**, **D** Coimmunoprecipitation assay reflecting the interaction between NRF1-Flag and HA-FBW7 (**C**) or HA-HRD1 (**D**) in HepG2 cells treated with various fatty acids. **E** Ubiquitination of NRF1 in HepG2 cells overexpressing NRF1-Flag and HA-Ub upon DHA stimulation, as indicated by a coimmunoprecipitation assay. **F** NRF1 expression in HepG2 cells overexpressing wild-type NRF1 or mutant NRF1 deficient in amino acids 1–103 treated with DHA. **G** Coimmunoprecipitation assay indicating the interaction between NRF1-Flag and HA-DDI2, which were overexpressed in HepG2 cells stimulated with various fatty acids.

Among various fatty acids, DHA prominently upregulated NRF1 expression, and our study delved into the specific molecular mechanisms through which DHA regulated NRF1 expression. Firstly, HepG2 cells were treated with cyclohexane (CHX) to inhibit protein synthesis, which reflected the rate of NRF1 degradation. NRF1 exhibited a short half-life of approximately 15 min under physiological conditions, with the full-length form of NRF1 being predominantly degraded. However, treatment with DHA significantly extended the half-life of NRF1 to 40 min (Fig. 4A), suggesting that DHA stabilized NRF1 by inhibiting its degradation. We next sought to elucidate the specific degradation pathway through which DHA promoted the accumulation of NRF1. Notably, treatment with the classical proteasome inhibitor MG132 reversed the DHA-induced increase of NRF1 protein levels (Fig. 4B), whereas treatment with the lysosomal inhibitor chloroquine (CQ) did not alter the effect of DHA (Fig. S2C). Since NRF1 is known to be degraded via the UPS, we focused on three previously reported E3 ubiquitin ligases potentially responsible for the DHA-mediated stabilization of NRF1 [20, 21]. As indicated by coimmunoprecipitation assays, the interactions between NRF1 and its cytoplasmic E3 ligases FBW7 (an adaptor for the Skp1–Cul1–F-box protein ubiquitin ligase complex) and HRD1 (an ER-associated degradation ubiquitin ligase) were impaired by the addition of DHA (Fig. 4C, D). However, the interaction between NRF1 and its nuclear E3 ligase β-TrCP, an adaptor for the SCF ubiquitin ligase complex, remained unaffected by DHA (Fig. S2D). As anticipated, the ubiquitination of NRF1 was also inhibited by DHA (Fig. 4E).

We further sought to ascertain the subcellular location where DHA affected NRF1 degradation. Therefore, we generated an N-terminal-deficient mutant of NRF1 (Δ1–103), representing the active form of NRF1 localized in the nucleus. We observed that DHA selectively upregulated the full-length form of NRF1 but had no effect on the active form (Fig. 4F), suggesting that DHA specifically inhibited the degradation of NRF1 in the cytoplasm rather than the nucleus. Given that DHA promoted nuclear translocation of NRF1, we assessed the cleavage of NRF1 and found that DHA enhanced the cleavage of NRF1 by the aspartic protease DNA damage-inducible 1 homolog 2 (DDI2), thereby facilitating the nuclear entry of the active form (Fig. 4G). These findings suggested that DHA inhibited the degradation of NRF1 by the UPS at the ER membrane rather than in the nucleus.

### NRF1 expression was increased by DHA in the context of MASH

We fed mice an HFD containing 4% DHA for 20 weeks to further understand the critical regulation of NRF1 by DHA in the context of MASH. Consistent with previous studies, DHA feeding dramatically mitigated hepatic steatosis, balloon degeneration of hepatocytes, liver damage and inflammation triggered by the HFD (Fig. S3A–D). Oxidative stress was also alleviated by the addition of DHA, as evidenced by the significant downregulation of hepatic malondialdehyde (MDA), one of the major end products of lipid peroxidation reactions (Fig. S3E). We subsequently detected NRF1 expression in liver tissues. Immunofluorescence, immunohistochemistry and immunoblotting revealed reduced NRF1 expression in HFD-fed mice, which was effectively reversed by the addition of DHA to the diet (Fig. S3F–H). Notably, DHA preferentially upregulated the expression of NRF1 in the pericentral zone compared to the intermediate and periportal zones, which coincided with the zone exhibiting the most significant amelioration of hepatic steatosis (Fig. S3G). As expected, DHA increased mRNA levels of proteasome subunits, whereas these genes were downregulated in HFD-induced MASH (Fig. S3I). Taken together, these data demonstrated that DHA enhanced the expression of NRF1 in the context of MASH and protected the liver against MASH progression.

Given the ubiquitous expression of NRF1 across multiple organs (Human Protein Atlas, Fig. S4A), we investigated whether DHA mediated NRF1 upregulation in tissues beyond the liver. In mice fed with an HFD containing DHA, we also observed the significant upregulation of NRF1 expression in white adipose tissue (Fig. S4B), which was further confirmed in human SW872 adipocytes (Fig. S4C). Despite the systemic effects on NRF1 expression, DHA supplementation specifically reduced liver weight without alteration in white adipose tissue mass and body weight (Figs. S4D and S4E). Our results suggested that while DHA modulated the expression of NRF1 in multiple tissues, its therapeutic benefits in MASH appeared to be primarily mediated through hepatic-specific effects.

### The absence of hepatic NRF1 reversed the protective effect of DHA against MASH

**Fig. 5:**
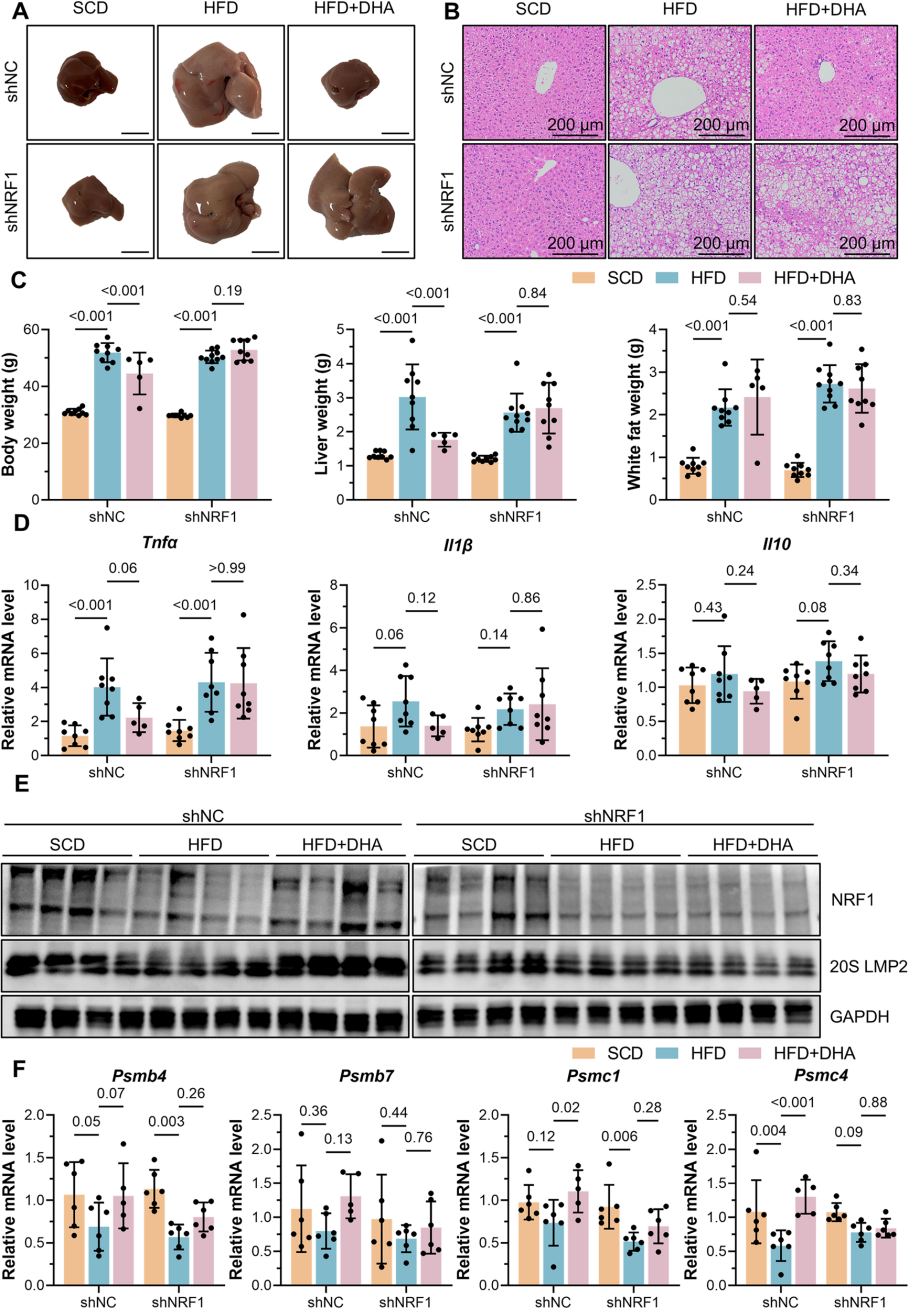
Liver-specific knockdown of NRF1 reversed the protective effect of DHA against MASH. **A** Gross observation of livers from WT and shNRF1 mice fed an SCD, pure HFD or HFD supplemented with 4% DHA for 20 weeks. Scale bar, 10 mm. **B** H&E staining of liver tissues from the abovementioned mice. **C** Body weight, liver weight and white fat weight of the abovementioned mice (shNC, SCD, *n* = 9; shNC, HFD, *n* = 9; shNC, HFD + DHA, *n* = 5; shNRF1, SCD, *n* = 9; shNRF1, SCD, *n* = 10; shNRF1, HFD + DHA, *n* = 9). **D** The gene expression of inflammatory factors (*Tnfα*, *Il1β* and *Il10*) in livers of the abovementioned mice (shNC, HFD + DHA, *n* = 5; *n* = 8 for other groups). **E** Western blot analysis of NRF1 and LMP2 expression in livers of the abovementioned mice (*n* = 4 for each group). **F** The gene expression of proteasome subunits (*Psmb4*, *Psmb7*, *Psmc1*, and *Psmc4*) in livers of the abovementioned mice (shNC, HFD + DHA, *n* = 5; *n* = 6 for other groups). Two-way ANOVA was used for the statistical analyses in (**C**, **D**, and **F**).

To further elucidate the significance of NRF1 in DHA-mediated protective effect against MASH, we generated mice with AAV8-driven knockdown of NRF1, specifically in liver tissues, and then fed the mice an HFD alone or the HFD containing 4% DHA for 20 weeks. Through a gross examination of livers, H&E staining of hepatic tissues and a record of liver weights, we found that the addition of DHA protected the liver against HFD-induced steatosis and liver injury, which was reversed by liver-specific NRF1 knockdown (Fig. 5A–C). In addition, inflammation in liver tissues was ameliorated by DHA in WT mice but not in NRF1-knockdown mice, as indicated by the gene expression of inflammatory factors (*Tnfα*, *Il1β* and *Il10*) (Fig. 5D). We then assessed proteasome abundance and found that both protein and mRNA levels of proteasome subunits were increased by addition of DHA, whereas liver-specific knockdown of NRF1 inhibited the promoting effect of DHA (Fig. 5E, F). These results suggested that the increase in proteasome abundance induced by DHA in MASH relied on hepatic NRF1.

To further validate our findings, we subsequently conducted in vitro assays in NRF1 knockout AML12 cells (Fig. S2A). In the FFA-induced cellular MASH model, the deficiency of NRF1 reversed the protective effect of DHA, as evidenced by increased lactate dehydrogenase (LDH) release into the supernatant, reduced cell viability assessed by CCK-8 assay, and increased propidium iodide (PI)-positive dead cells (Fig. S5A–C). To investigate the contribution of proteasome activity to DHA-mediated protective effect against liver injury, we treated cells with MG132. Notably, MG132 reversed the ameliorative effect of DHA, as indicated by re-elevated LDH levels in the supernatant (Fig. S5D). Taken together, these findings demonstrated that the upregulation of NRF1 by DHA explained its protective effect against liver injury in MASH, particularly through the increase in proteasome abundance.

### NRF1 ameliorated liver injury in MASH by inhibiting ER stress

**Fig. 6:**
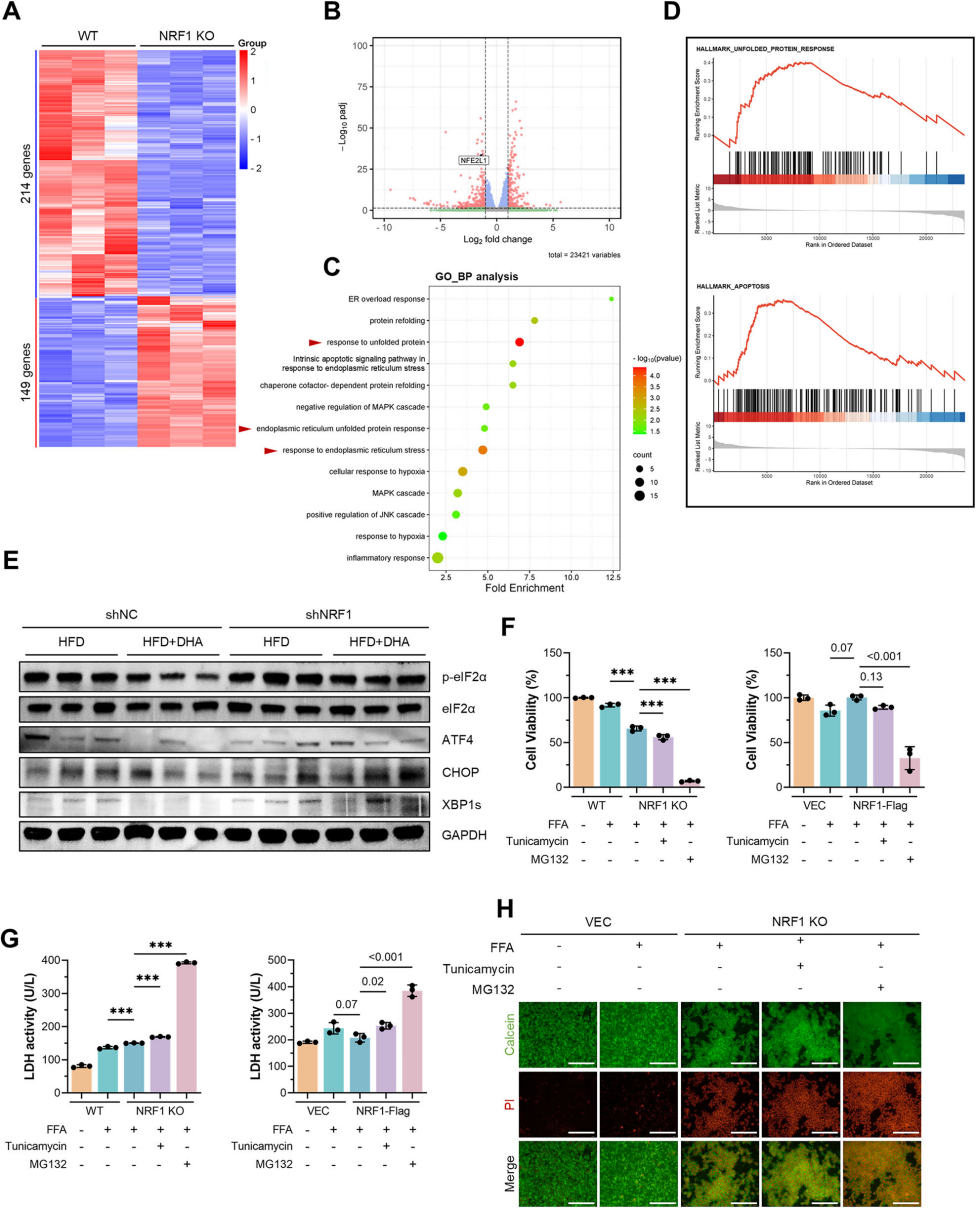
NRF1 ameliorated liver injury by inhibiting ER stress. **A** Heatmap depicting the z scores of significantly differentially expressed genes identified via RNA-seq analysis of AML12 cells with NRF1 deficiency. **B** Volcano plot showing significantly differentially expressed genes (DEGs) and their fold changes in WT and NRF1-deficient AML12 cells. **C** GO pathway enrichment analysis of DEGs identified via RNA-seq. **D** GSEA of DEGs identified via RNA-seq. **E** Western blot analysis of liver tissues from mice fed an HFD alone or the HFD supplemented with 4% DHA exhibiting the expression of p-eIF2α, eIF2α, ATF4, CHOP and XBP1s. **F**, **G** Cell viability evaluated by a CCK-8 assay (**F**), and the LDH level in the supernatant (**G**) of AML12 cells with altered NRF1 expression after stimulation with FFAs together with tunicamycin or MG132. **H** Calcein/PI staining of WT and NRF1-deficient AML12 cells treated with FFA together with tunicamycin or MG132. Scale bar, 200 μm. The data were plotted as the means ± SEMs. One-way ANOVA was used for the statistical analyses in (**F**, **G**). *** *P* < 0.001.

To elucidate the underlying mechanisms by which NRF1 attenuated MASH progression, we studied NRF1-dependent gene expression and performed a transcriptome analysis of FFA-treated WT and NRF1-deficient AML12 cells. RNA-seq analysis identified 363 differentially expressed genes (DEGs), comprising 149 upregulated and 214 downregulated transcripts in NRF1-knockout cells (Fig. 6A). The significant reduction in NRF1 transcription, evident in the volcano plot, validated the reliability of our RNA-seq data (Fig. 6B). Moreover, GO enrichment analysis suggested that the most significantly enriched pathways were involved in the response to ER overload stress, the apoptotic signalling pathway in response to ER stress or the response to unfolded proteins (Fig. 6C). Gene set enrichment analysis (GSEA) further confirmed marked dysregulation of unfolded protein response and apoptosis pathways (Fig. 6D). Therefore, our transcriptome analysis established a robust and highly reproducible link between NRF1-dominant proteasome abundance and ER stress, particularly the ER-associated protein degradation (ERAD) pathway.

Unfolded protein response signalling and chronic ER stress triggered by lipotoxic cellular damage have been shown to drive cellular death and inflammation, contributing to the progression of MASH [22–24]. Here, we showed that ER stress was ameliorated by DHA, as indicated by the increased phosphorylation of eIF2α and downstream activation of ATF4 and CHOP in mice fed an HFD for 20 weeks. However, NRF1 deficiency reversed the beneficial effect of DHA on ER stress (Fig. 6E). To determine the role of ER stress in the protective effect of NRF1 against MASH, we treated AML12 cells with the ER stress inducer tunicamycin. The CCK-8 assay results, LDH levels in the supernatant and PI staining results all indicated that both tunicamycin and MG132 treatment reversed the protective effect of NRF1 against FFA-induced liver injury (Fig. 6F–H). These results suggested that NRF1 alleviated liver injury in MASH through the inhibition of ER stress.

### NRF1 alleviated oxidative stress triggered by ER stress in MASH

**Fig. 7:**
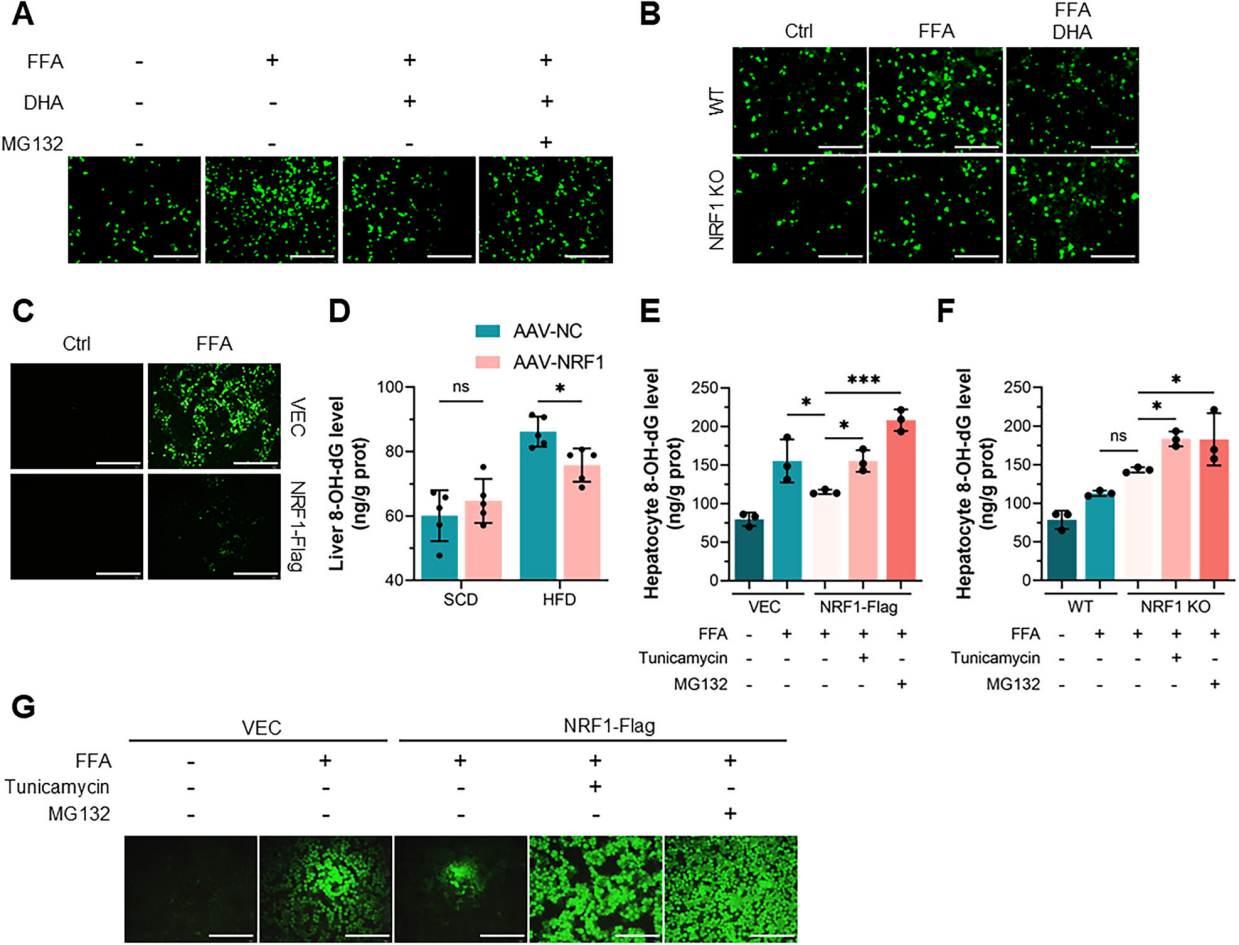
NRF1 alleviated oxidative stress in MASH by inhibiting ER stress. **A** Representative image of DCFH-DA staining of intracellular ROS in AML12 cells treated with FFAs together with DHA and MG132. Scale bar, 200 μm. **B** Representative images of DCFH-DA staining of intracellular ROS in WT and NRF1-deficient AML12 cells treated with FFAs together with DHA. Scale bar, 200 μm. **C** Representative images of DCFH-DA staining of intracellular ROS in AML12 cells transfected with NRF1-Flag and subjected to FFAs treatment. Scale bar, 200 μm. **D** Detection of 8-OH-dG levels in liver tissues from WT and NRF1-overexpressing mice fed the SCD or HFD for 20 weeks. **E**, **F** Detection of 8-OH-dG levels in AML12 cells with altered NRF1 expression upon stimulation with FFAs together with tunicamycin or MG132. **G** Representative images of DCFH-DA staining of intracellular ROS in AML12 cells transfected with NRF1-Flag and stimulated with FFAs together with tunicamycin or MG132. Scale bar, 200 μm. The data were plotted as the means ± SEMs. Two-way ANOVA in (**D**) and one-way ANOVA in (**E**, **F**) were used for the statistical analyses. ns: not significant, * *P* < 0.05, and *** *P* < 0.001.

Increased evidence underscored the critical role of oxidative stress in MASH pathogenesis induced by metabolic stresses [25–27]. Our study revealed that DHA significantly reduced the accumulation of cellular reactive oxygen species (ROS) induced by FFAs, which was reversed by proteasome inhibition (Fig. 7A). Additionally, the absence of NRF1 abolished the protective effect of DHA against oxidative stress, as indicated by both cellular ROS level and superoxide dismutase (SOD) activity (Figs. 7B and S5E). In contrast, overexpression of NRF1 alleviated oxidative stress in MASH, as indicated by reduced ROS levels and decreased levels of the oxidation product 8-hydroxy-2’-deoxyguanosine (8-OH-dG) (Fig. 7C, D). Considering the crucial role of ER stress and unfolded protein response (UPR) signalling in the protective effect of NRF1 against MASH, we treated AML12 cells with either tunicamycin or MG132. The detection of both ROS level and 8-OH-dG level suggested that tunicamycin or MG132 treatment reversed the protective effect of NRF1 against oxidative stress (Fig. 7E–G). Taken together, these results indicated that NRF1 mitigated oxidative stress triggered by ER stress and the UPR in MASH.

### NRF1, but not NRF2, ameliorated liver injury in MASH by increasing proteasome abundance

While both NRF1 and nuclear factor erythroid-derived 2-related factor 2 (NRF2, also known as NFE2L2) belong to the CNC family of transcription factors and regulate ARE-dependent genes, our investigation revealed key functional distinctions between these paralogs in MASH pathogenesis. First, we found that NRF1 rather than NRF2 governed the transcription of proteasome subunits (Fig. S6A). Second, NRF2 expression remained unaffected by DHA treatment, in contrast to NRF1 expression (Fig. S6B). Third, the overexpression of NRF2 failed to mitigate FFA-induced hepatocyte injury, as indicated by the detection of LDH levels in the supernatant (Fig. S6C). Taken together, these findings suggested that NRF1 played a distinct role from that of NRF2 in the transcriptional regulation of proteasome subunits and in the DHA-mediated protective effect against liver injury in MASH.

## Discussion

This study aimed to elucidate the key molecular mechanisms underlying the progression of MASH, laying a foundation for the development of novel therapeutic strategies. We focused on the transcription factor NRF1, which is a central regulator of proteasome biogenesis. Here, we demonstrated a pivotal pathophysiological role of NRF1 in the progression of MASH, where its expression was downregulated in both experimental models and clinical specimens, accompanied by impaired proteasomal activity. Furthermore, liver-specific overexpression of NRF1 dramatically increased proteasome abundance and ameliorated MASH, as evidenced by the alleviation of steatosis, mitigation of liver damage, decreased infiltration of inflammatory cells and inhibition of hepatic fibrosis. Taken together, these findings provided compelling experimental evidence to establish NRF1 as a master regulator that alleviated MASH primarily through enhancing the proteasome-mediated degradation of misfolded and damaged proteins.

Previous studies have revealed that the absence of NRF1 affected cholesterol, triglyceride, and glucose metabolism as well as attenuated defended against ROS, proteotoxicity and impaired autophagy. Particularly, deficiency of NRF1 resulted in apoptosis, steatosis and spontaneous development of tumors in the mouse liver. However, the mechanisms underlying these phenotypes are not clear. Herein, we proposed that NRF1 alleviated the steatosis, liver damage, inflammation and fibrosis of MASH by inhibiting ER stress in hepatocytes. Admittedly, ER plays a pivotal role in maintaining proteostasis, where accumulation of unfolded proteins triggers ER stress and initiates the UPR pathway [28]. Severe and unresolved ER stress causes persistent UPR activation, resulting in cell death, inflammasome activation and progressive liver damage [29, 30]. NRF1 inhibited ER stress by enhancing UPS activity to facilitate the clearance of unfolded proteins, thereby reducing oxidative stress and preventing cell death.

Notably, although NRF1 is ubiquitously expressed across cell types and governs the transcription of proteasome subunits, the regulation of its expression and activation is poorly understood. In this study, we investigated DHA, a long-chain PUFA with established hepatoprotective properties, for its previously unrecognized role in proteasome-mediated protein degradation.

In terms of DHA, the most thoroughly studied effect was its anti-inflammatory property through suppression of proinflammatory cytokines and enhancement of antioxidant defenses [31]. G protein-coupled receptor 120 (GPR120), a functional receptor for ω-3 PUFA, could inhibited TLR and TNF-α inflammatory signalling pathway when responded to DHA stimulation in pro-inflammatory macrophages and mature adipocytes [32, 33]. Structural studies identified specific aromatic residues that recognized the double C‒C bonds in PUFAs [34]. However, the protective effect of DHA against liver damage was poorly studied. Herein, we firstly suggested that DHA could ameliorate liver damage through upregulation of NRF1.

Further investigation into the regulatory mechanism revealed that DHA specifically prevented the ubiquitination and degradation of NRF1 at the ER membrane rather than in the nucleus, resulting in accumulation of full-length NRF1. In addition, DHA affected the interaction of NRF1 with its processing protease DDI2, thereby facilitating the activation and nuclear translocation of NRF1. This raises an intriguing question regarding the selective upregulation of cytoplasmic NRF1 by DHA. Considering that DHA is a crucial component of cellular membrane phospholipids, it has been reported to impact the function of membrane proteins by perturbing the stability and regularity of the cell membrane [35–37]. We propose a model where DHA incorporation into ER membrane phospholipids disrupts the regular arrangement of lipid bilayer due to the special three-dimensional shape of DHA; then, the full-length form of NRF1 at the ER membrane is perturbed and overturned from the ER lumen to the cytoplasm, where it can be recognized and cleaved by the cytoplasmic protease DDI2 to generate the active form of NRF1 [38, 39].

Our study elucidated the distinct features of NRF1 and NRF2, both members of the CNC family of transcription factors that activated ARE-driven genes. While these factors share certain characteristics, several lines of evidence demonstrate their nonredundant functions. First, a previous study showed that NRF1, rather than NRF2, was indispensable for embryonic development [40]. Second, NRF1 and NRF2 were discovered to be complementary genes that modulated cholesterol-associated fatty liver disease progression [41]. Metabolically, deficiency of NRF1, rather than NRF2, promoted distinct cellular adaptions, including enhanced glycolysis, reduced mitochondrial oxygen consumption, increased gluconeogenesis and activation of the pentose phosphate pathway [42, 43]. Moreover, despite their shared ability to bind ARE sequences, their downstream gene expression profiles were notably different [44, 45]. Several potential molecular mechanisms have been proposed. In terms of the protein structure, the N-terminal extension in NRF1 includes an additional 155-amino-acid polypeptide which is responsible for NRF1 being directed to the endoplasmic reticulum (ER), whereas NRF2 is located in the cytosol [46]. Considering that most of the DHA taken up by cells was converted into components of membrane phospholipids, we speculated that DHA interfered with membrane fluidity and lipid raft structure, thereby affecting the interaction between NRF1 and E3 ligases. This resulted in the accumulation of NRF1. However, NRF2 was located in cytosol that may not affected by DHA through alterant fluidity of the membrane. Totally, the distinct subcellular localization and function of NRF1 may partly explain its unique response to DHA treatment different from NRF2.

In conclusion, our study was the first to emphasize that reduced NRF1 expression and consequent impairment of proteasomal degradation were critical for driving MASH progression through exacerbated ER stress and subsequent irreversible oxidative damage. Moreover, we have identified a novel regulatory axis connecting DHA supplementation with NRF1 stabilization and proteasome activation, significantly advancing our understanding of cellular adaptive responses in MASH. These findings provided important insights in endogenous regulatory mechanism of NRF1 in disease pathogenesis. Our work not only elucidated fundamental aspects of NRF1 biology but also opened new therapeutic avenues for MASH treatment by targeting this previously unknown proteostatic pathway.

## Supplementary information

Unedited blot and gel image

Supplemental Materials

## Data Availability

The raw data for the article are available in the Supporting Data Values file.
