# Supplementary material for: NRF1 is upregulated by docosahexaenoic acid to ameliorate MASH through the inhibition of ER stress
Source: Cell Death Dis. 2026 Jan 16;17(1):47. doi: 10.1038/s41419-025-08139-1 (PMC12811613; doi:10.1038/s41419-025-08139-1)

Uncropped gels for Western Blots in Figure 1

Figure 1A

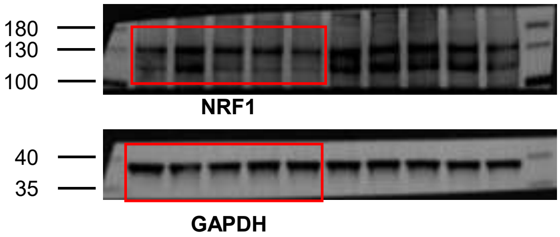

Figure 1B

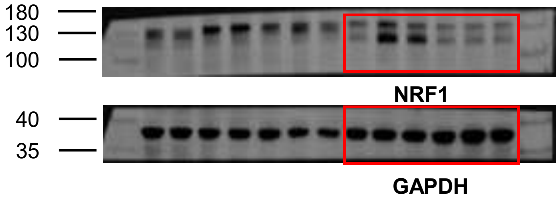

Figure 1D

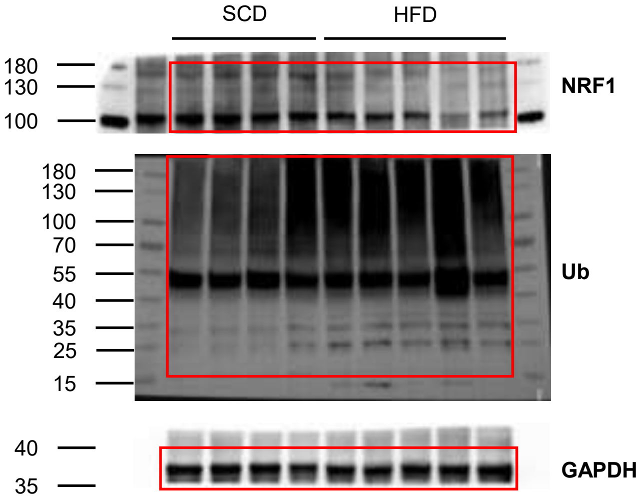

Figure 1F

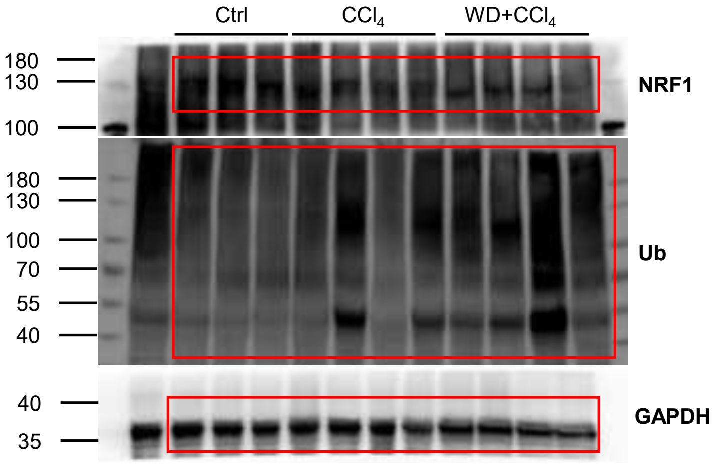

Uncropped gels for Western Blots in Figure 2

Figure 2F

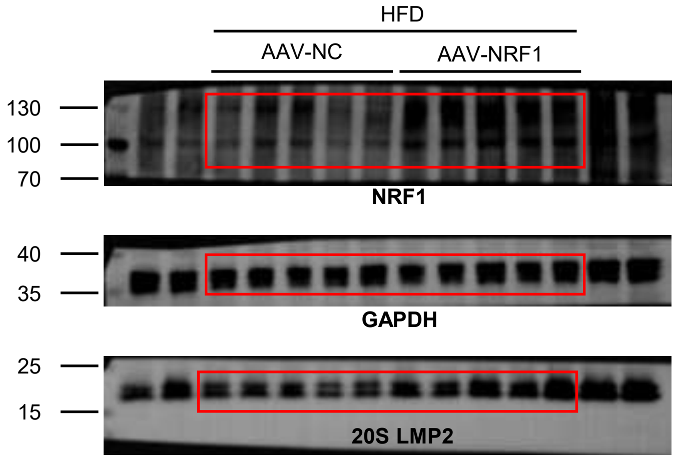

Uncropped gels for Western Blots in Figure 3

Figure 3A

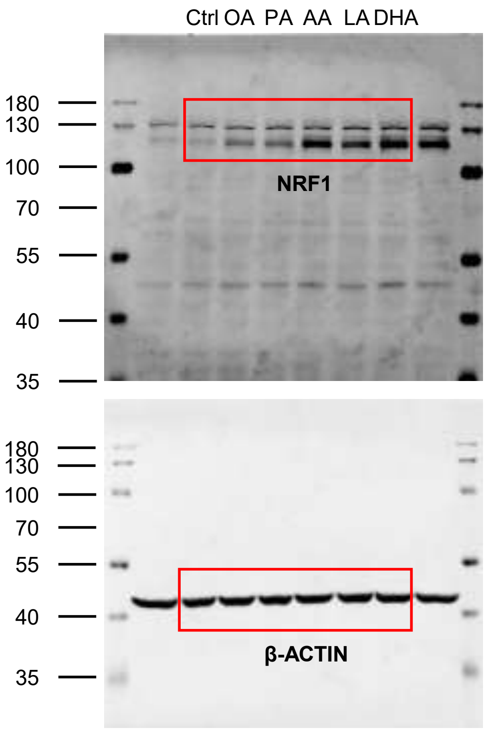

Figure 3B

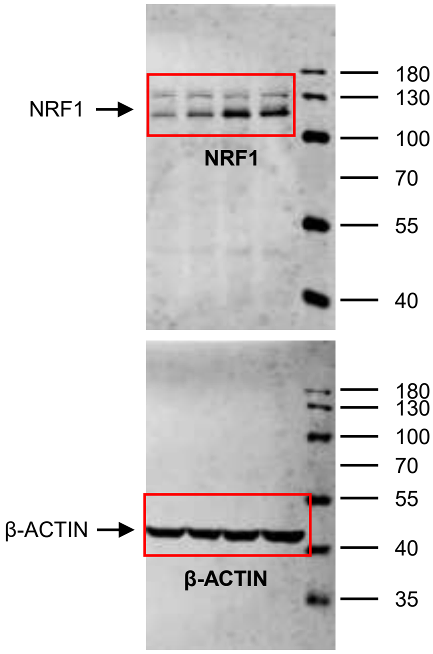

Figure 3E

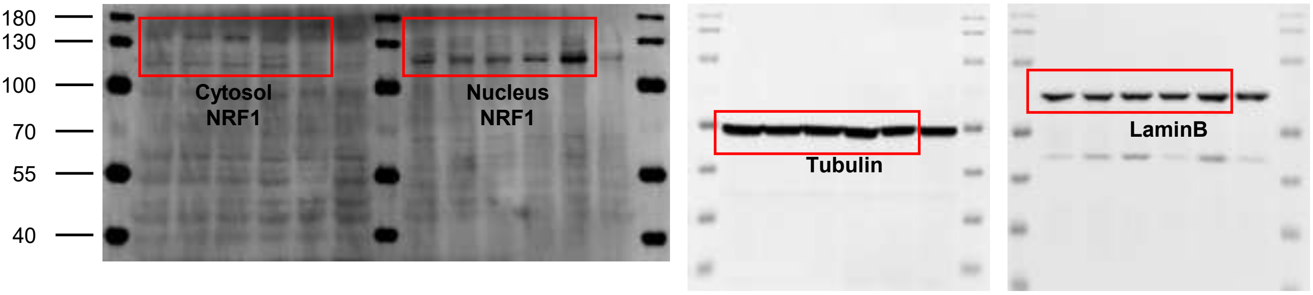

Uncropped gels for Western Blots in Figure 4

Figure 4A

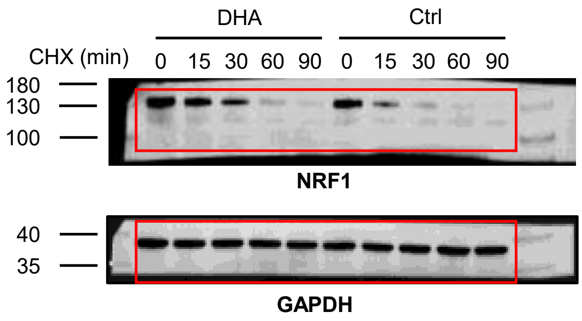

Figure 4B

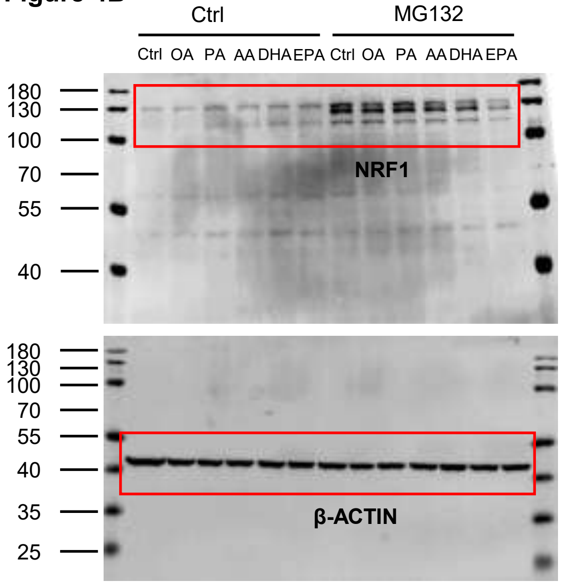

Figure 4C

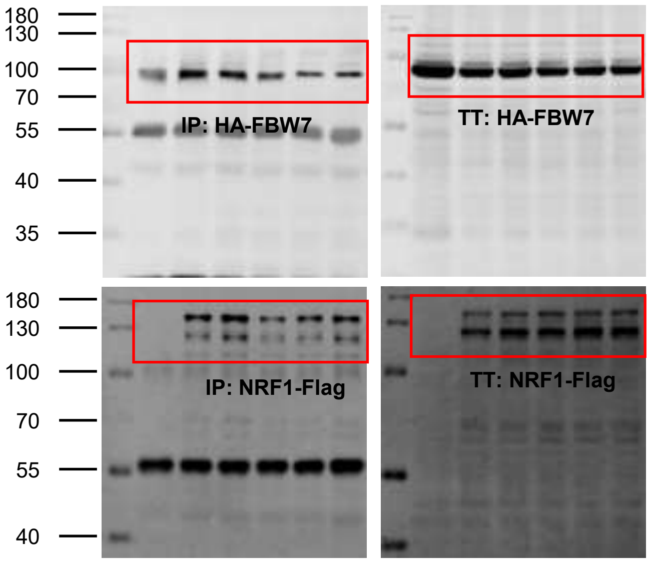

Figure 4D

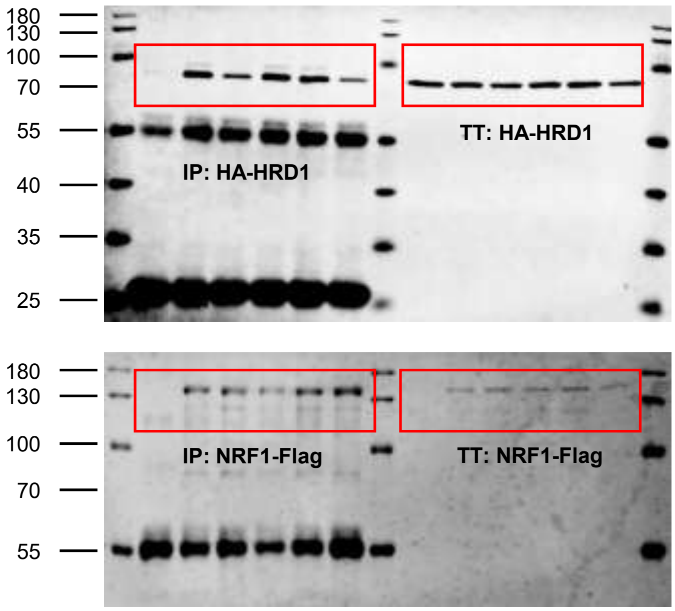

Uncropped gels for Western Blots in Figure 4

Figure 4E

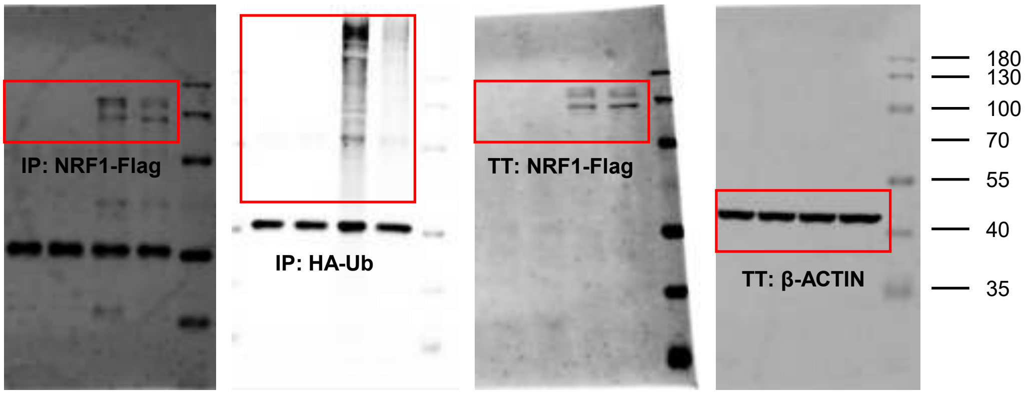

Figure 4F

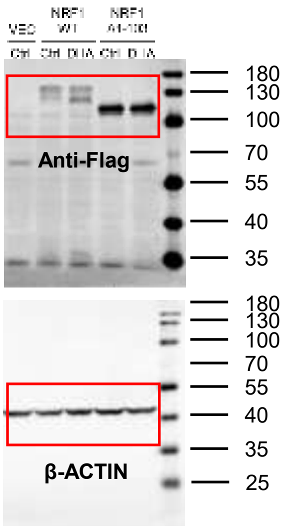

Figure 4G

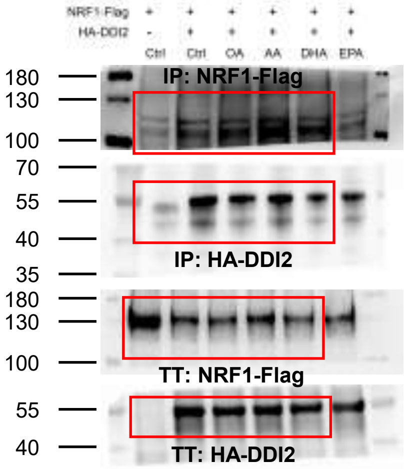

Uncropped gels for Western Blots in Figure 5

Figure 5E

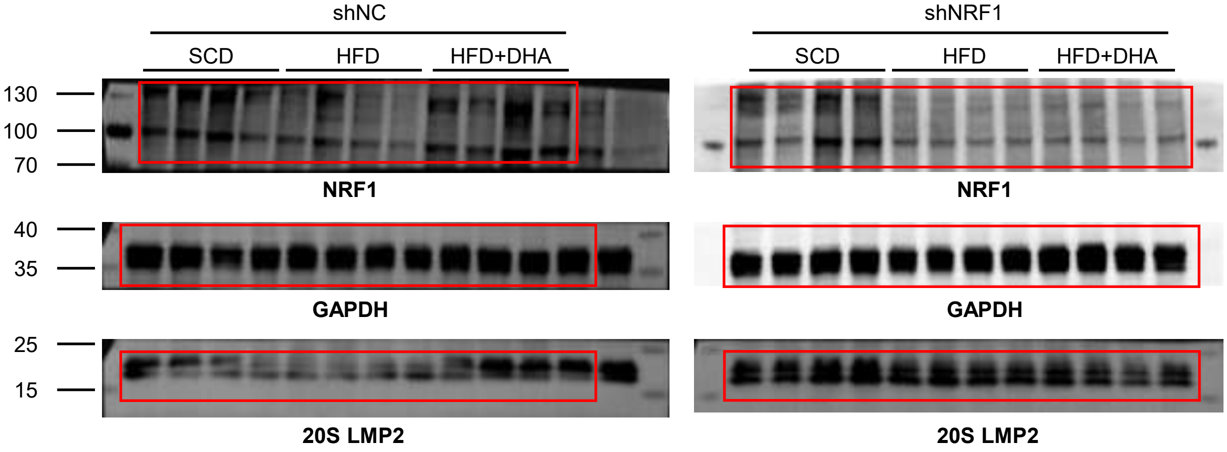

Uncropped gels for Western Blots in Figure 6

Figure 6E

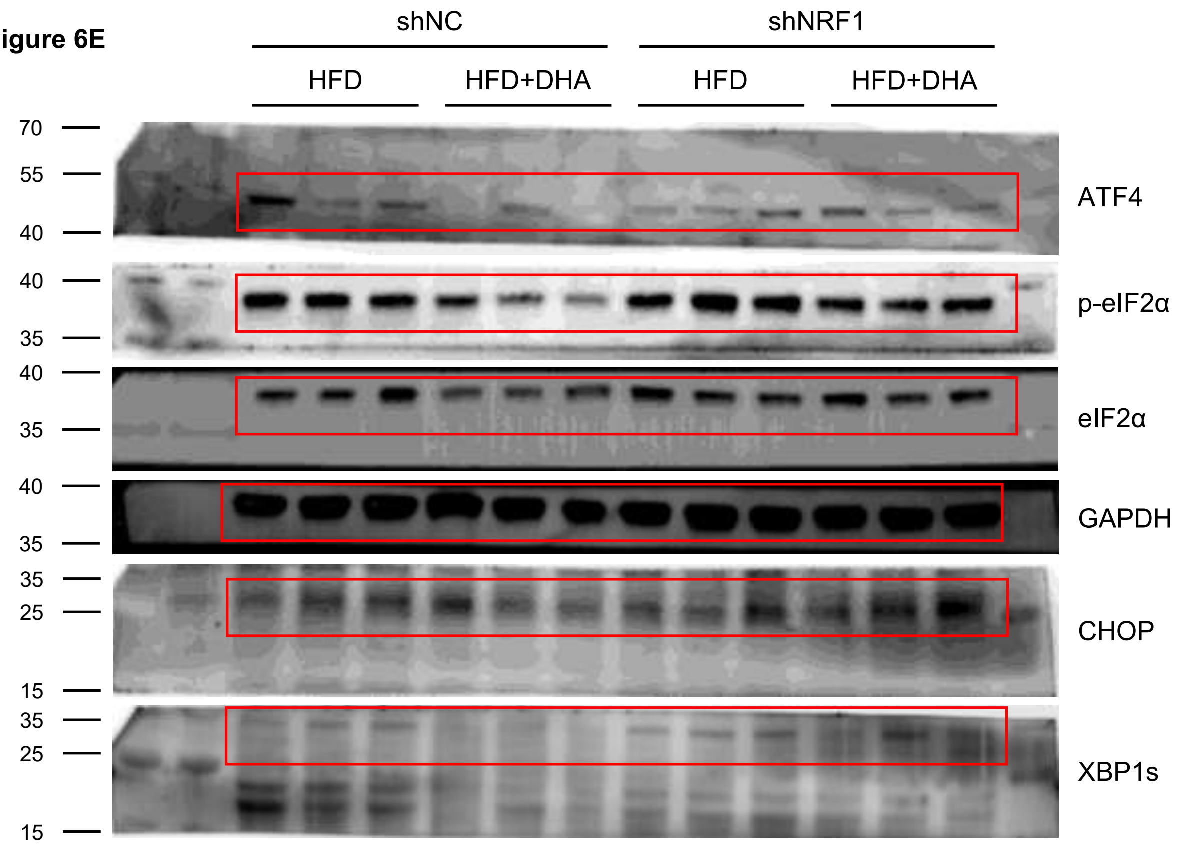

Uncropped gels for Western Blots in Figure S2

Figure S2A

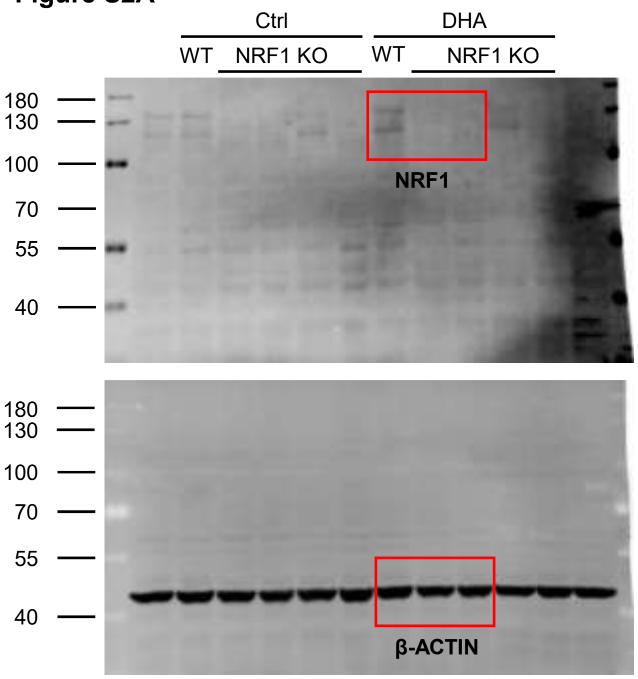

Figure S8B

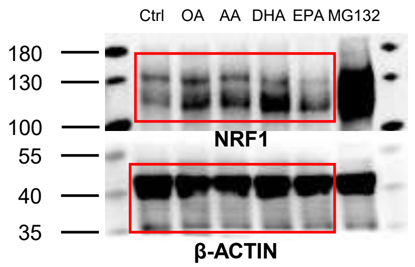

Figure S2C

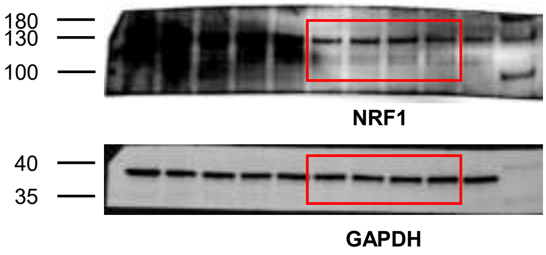

Figure S2D

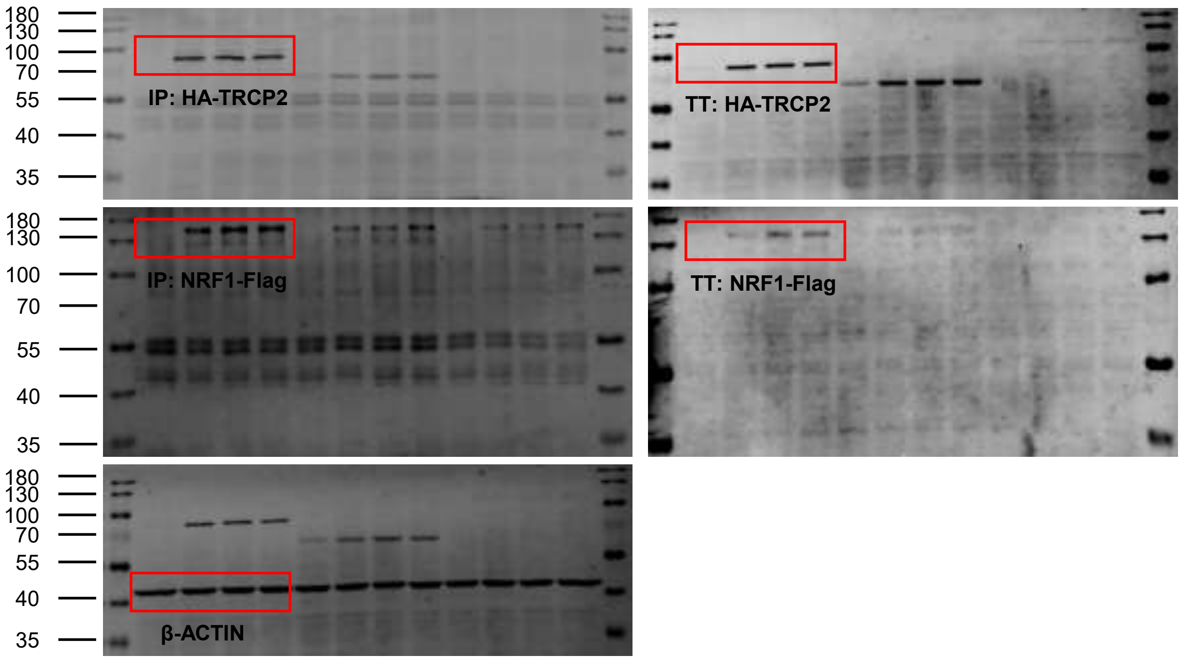

Uncropped gels for Western Blots in Figure S3

Figure S3H

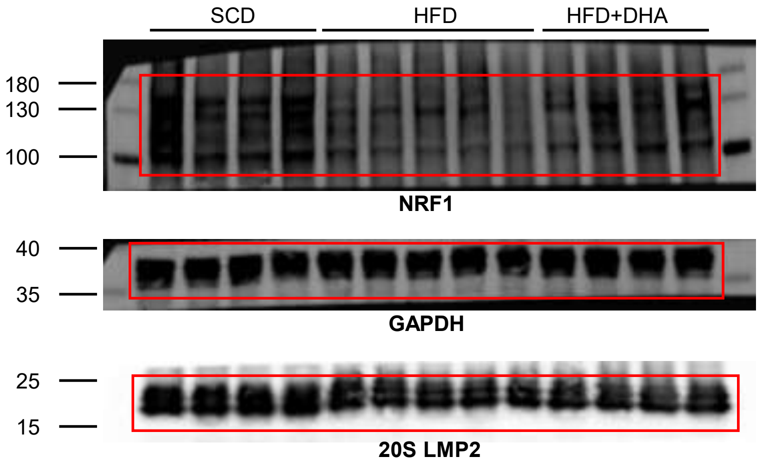

Uncropped gels for Western Blots in Figure S4

Figure S4B

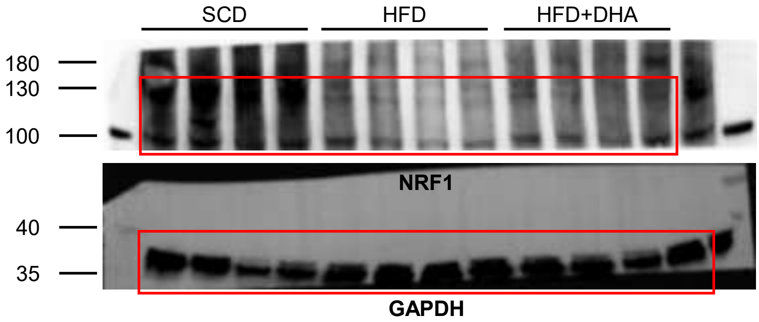

Figure S4C

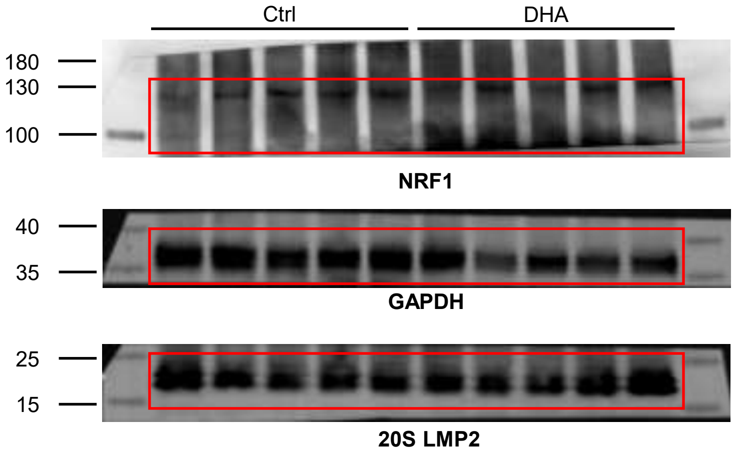

Uncropped gels for Western Blots in Figure S6

Figure S6B

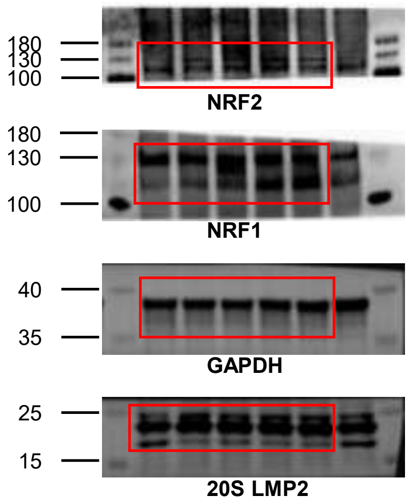

Supplement: Supplementary file 1 — Unedited blot and gel image [file 41419_2025_8139_MOESM1_ESM.pdf]
