# Supplementary material for: NRF1 is upregulated by docosahexaenoic acid to ameliorate MASH through the inhibition of ER stress
Source: Cell Death Dis. 2026 Jan 16;17(1):47. doi: 10.1038/s41419-025-08139-1 (PMC12811613; doi:10.1038/s41419-025-08139-1)
Supplement: Supplementary file 2 — Supplemental Materials [file 41419_2025_8139_MOESM2_ESM.docx]

**Supplemental Materials for**

**NRF1 is upregulated by docosahexaenoic acid to ameliorate MASH through the inhibition of ER stress**

Lin *et al*

*Correspondent author: Chengfu Xu, xiaofu@zju.edu.cn

**The PDF file includes:**

Supplemental Methods

Tables S1 to S4

Figures S1 to S5

**Supplemental Methods**

**Quantification of proteasome activity**

Quantification of proteasome activity was performed using a 20S proteasome activity kit (SKT-133, StressXpress) according to the operating instruction. Briefly, drug-treated cells were incubated with 1 mM working solution of proteasome substrate Suc-LLVY-AMC for more than 2 h at 37 °C without light and fluorescence was measured using microplate reader (490 nm excitation wavelength, 525 nm emission wavelength).

**Quantification of lactate dehydrogenase (LDH), malondialdehyde (MDA), superoxide dismutase (SOD) and** **8-hydroxy-2'-deoxyguanosine (8-OH-dG)**

LDH in the cell culture supernatant was detected using LDH cytotoxicity assay kit (C0016, Beyotime) according to its operating instruction. MDA level of liver tissue was assessed via MDA assay kit (A003-4-1, Nanjing Jiancheng Bioengineering Institute) after grinding and crushing tissues in RIPA lysis buffer. SOD activity of adherent cells was tested via SOD assay kit (WST-1 method) (A001-3-2, Nanjing Jiancheng Bioengineering Institute) after lysing cells by ultrasound in PBS. 8-OH-dG in liver tissues and cultured cells was detected via 8-hydroxydeoxyguanosine Assay Kit (H165-1-2, Nanjing Jiancheng Bioengineering Institute).

**Immunohistochemistry (IHC)**

Immunohistochemistry assays were performed, as previously described. The primary antibodies are listed in Table S2. More than three fields of each sample were randomly taken for quantitative assessment using Image J software.

**Co-immunoprecipitation**

Cells were lysed with NP-40 buffer (50 mmol/L HEPES, 150 mmol/L NaCl, 10% glycerol, 1% NP-40, 1 mmol/L EDTA, 1 mmol/L EGTA, pH 7.4) supplemented with protease inhibitor cocktail and phosphatase inhibitor. Following sonication, the lysate was incubated overnight at 4 °C with specific antibodies, followed by addition of protein A or protein G beads for precipitation. After three washes with lysis buffer, the protein was eluted from beads with SDS loading buffer at 70 °C for 10 min.

For the ubiquitination assay, cells were lysed with urea buffer (0.05 M Tris-HCl, 0.1 M NaH2PO4, 8 M urea, 0.04 M imidazole, 0.5% CHAPS) and immunoprecipitated as mentioned above.

**Dual luciferase reporter gene assay**

Dual luciferase reporter gene assay was performed in HepG2 cells to verify DHA-induced transcriptional activation of NRF1. The triple antioxidant response element (ARE) from promoter region of human proteasome subunit PSMA4, including wild type and mutant, were separately cloned into pGL3-basic vector with inserted sequences as follows:

5'-cgagccgtgggcacgaTGACTCTGCccgcctcctctgagccgtgggcacgTGACTCTGCAccgcct

cctctgagccgtgggcacgaTGACTCTGCA ccgcctcctctg-3' (wild type);

5'-cgagccgtgggcacgaTGACTCTAAAccgcctcctctgagccgtgggcacgTGACTCTAAAccgc

ctcctctgagccgtgggcacgaTGACTCTAAAccgcctcctctg-3' (mutant).

Briefly for luciferase assays, HepG2 cells grown on 6-well plates were transfected with indicated plasmids using lipofectamine 3000 (Invitrogen). pRL-TK vector expressing renilla luciferase was co-transfected to normalize the luciferase activity. Cell lysates were prepared using passive lysis buffer and assays were performed with Dual-Luciferase Reporter Assay System (E1910, Promega) according to the manufacturer’s instructions.

**CCK-8 assay**

The CCK-8 assay was performed to assess cell viability using Cell Counting Kit-8 (HY-K0301, MCE). Hepatic cells grown on 96-well plates were incubated with 10 μL CCK-8 buffer for more than 30 min at 37 °C, followed by detection with a microplate reader at 450 nm.

**Western blotting**

Briefly, cells or tissues were lysed with RIPA buffer supplemented with cocktail and phosphatase inhibitor (Sigma). The concentration of total protein was quantified with a BCA protein measurement kit (P0009, Beyotime). Proteins were separated by SDS-PAGE and transferred to PVDF membranes. The membrane was then blocked with 5% skim milk in TBST and immunoblotted with the primary antibody at 4 °C overnight, followed by incubation with HRP-conjugated secondary antibody for 1 h at room temperature. Primary antibodies are listed in Table S2. Finally, the protein was detected with an ECL light detection kit (Lianke Multi Sciences). GAPDH and β-ACTIN were appointed as the internal controls.

For nuclear and cytoplasmic separation assay, cells grown on 6-well plates were lysed and separated using Nuclear and Cytoplasmic Protein Extraction Kit (C510001, Sangon Biotech) according to manufacturer’s instructions. Extracted protein was then loaded for western blotting.

**Immunofluorescence staining, detection of ROS and calcein/PI staining**

Immunofluorescence staining was performed to demonstrate intracellular localization of NRF1. For fluorescence imaging of adherent cells, cells growing on glass coverslips were fixed with 4% paraformaldehyde (PFA) for 15 min at room temperature, permeabilized with 0.1% Triton X-100 for 10 min, blocked with 5% fetal bovine serum (FBS) for 1 h and immunoblotted with specific primary antibody and secondary antibody. The nucleus was labeled with DAPI. The confocal images were captured on Leica STELLARIS 5 confocal microscope.

For immunofluorescence staining of paraffin-embedded hepatic tissue sections (4 μm thickness), the histological slides were deparaffinized, rehydrated with ethanol, blocked of endogenous peroxidase activity with 3% H_2_O_2_ followed by antigen retrieval in citrate buffer. The slides were then blocked with 5% FBS and immunoblotted with specific primary antibody and secondary antibody.

Calcein/PI staining of adherent cells reflecting permeability of cell membrane was performed using calcein/PI cell viability/cytotoxicity assay kit (C2015S, Beyotime) according to manufacturer’s instructions.

**ROS detection**

ROS of hepatic cells was detected and exhibited using reactive oxygen species assay kit (S0033S, Beyotime). DCFH-DA with concentration of 1 µM was loaded into 1 × 10^6^ cells/100 μL, incubated at 37 °C for 30 min and washed thrice with 1× PBS. ROS was detected by confocal microscope or flow cytometer followed by collection of cells.

**Quantitative real-time PCR (qRT-PCR)**

The total RNA from incubated cells or liver tissues was extracted with RNA extraction kit (AG21017, Accurate Biotechnology Co. Ltd., Changsha, China) and reverse transcribed into cDNA with Evo M-MLV RT Master Mix (AG11706, Accurate Biotechnology). Quantitative PCR was conducted using a SYBR Green Premix Pro Taq HS qPCR Kit (AG11701, Accurate Biotechnology) and amplified with PCR amplifier (BIO-RAD CFX384). The Primer sequences are listed in Table S3 and S4.

**RNA-seq and data processing**

Total RNA was extracted from AML12 cells with RNA extraction kit. The purified RNA was then used for cDNA synthesis, adaptor ligation, and enrichment through low-cycle amplification, as per the instructions of the NEBNext® Ultra™ RNA Library Prep Kit for Illumina (NEB, Ipswich, MA). The resulting library products were evaluated using the Agilent 2200 TapeStation and Qubit® 2.0 (Thermo), then diluted to 10 pM for cluster generation on the paired-end flow cell, followed by sequencing (2×150 bp) on the HiSeq3000. Differential expression analysis was performed using DEseq, with read counts as input. Differentially expressed genes were selected based on a fold change > 2 and an adjusted *P* value < 0.05. These genes were further analyzed using gene ontology (GO) pathway enrichment analysis, gene set enrichment analysis (GSEA), and heatmap visualization. For both GO and GSEA enrichment analyses, a *P* value < 0.05 was set as the threshold for significant gene set enrichment.

**Supplemental Tables**

**Table S1.** Formula of rodent high-fat diet supplemented with 4% DHA


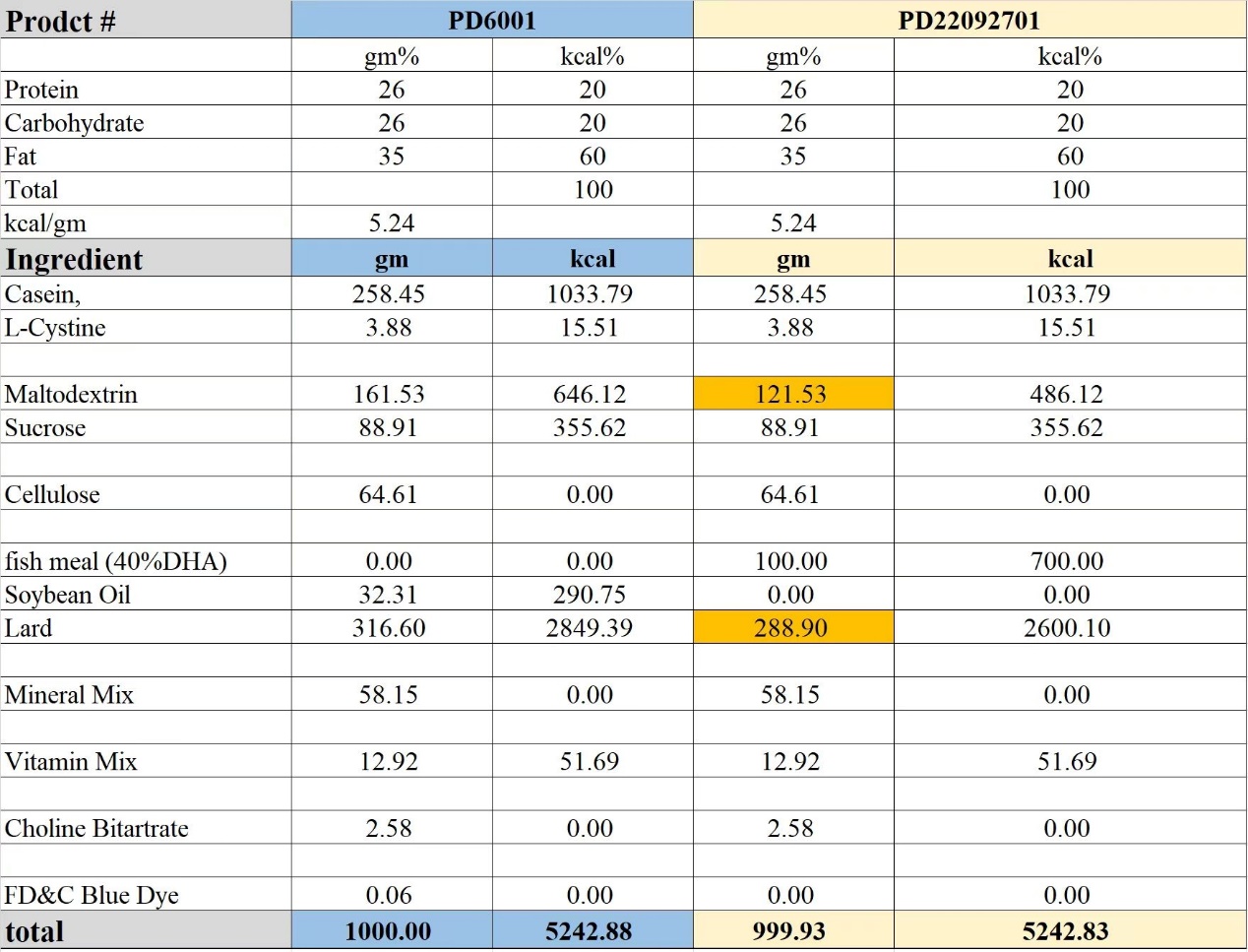


**Table S2.** Antibodies

| **Name** | **Supplier** | **Cat no.** |
| --- | --- | --- |
| TCF11/NRF1 Rabbit mAb | Cell Signaling Technology | 8052 |
| Anti-Nrf2 antibody | Abcam | Ab137550 |
| Anti-Proteasome 20S LMP2 antibody | Abcam | Ab242061 |
| Anti-ATF-4 antibody | Abcam | Ab31390 |
| CHOP (D46F1) Rabbit mAb | Cell Signaling Technology | 5554 |
| XBP1 Rabbit mAb | ABclonal | A25319 |
| Phospho-PERK (Thr980) (16F8) Rabbit mAb | Cell Signaling Technology | 3179 |
| PERK Rabbit pAb | ABclonal | A21957 |
| Phospho-eIF2α (Ser51) Antibody | Cell Signaling Technology | 9721 |
| eIF2α Rabbit mAb | ABclonal | A21221 |
| Ubiquitin Mouse mAb | Cell Signaling Technology | 3936 |
| Anti-HA-tag mAb | MBL | M180-3 |
| Anti-DDDDK-tag mAb | MBL | M185-3L |
| β-Tubulin Mouse mAb | ABclonal | AC021 |
| Beta Actin Monoclonal antibody | proteintech | 66009-1-lg |
| HRP-conjugated GAPDH Mouse mAb | ABclonal | AC035 |

**Table S3.** Primer sequences (homo sapiens) in qRT-PCR

| Primer | Forward (5’ to 3’) | Reverse (5’ to 3’) |
| --- | --- | --- |
| *NFE2L1* | CATTCTGCTGAGTTTGATTGGGG | TTGTGGAACTGGGTCTGAGTAT |
| *NFE2L2* | TCAGCGACGGAAAGAGTATGA | CCACTGGTTTCTGACTGGATGT |
| *PSMA7* | CTGTGCTTTGGATGACAACG | CGATGTAGCGGGTGATGTACT |
| *PSMB4* | CTCGTTTCCGCAACATCTCT | TGTCCATCTCCCAGAAGCTC |
| *PSMB7* | TGCAAAGAGGGGATACAAGC | GCAACAACCATCCCTTCAGT |
| *PSMC1* | TTCCGAGTTGCTGAAGAACA | ATCCATCCAACTGGTTCAGC |
| *PSMC4* | GGAAGACCATGTTGGCAAAG | AAGATGATGGCAGGTGCATT |
| *PSMD1* | GGGGACCTCTTCAATGTCAA | GCCTTCCAATCTCTGGTCAA |
| *PSMD12* | GTGCGCGACTGACTAAAACA | TAGGCAGAGCCTCATTTGCT |
| *NQO1* | AGCCCAGATATTGTGGCTGA | CGGAAGGGTCCTTTGTCATA |
| *GAPDH* | GGTGGTCTCCTCTGACTTCAACA | GTTGCTGTAGCCAAATTCGTTGT |

**Table S4.** Primer sequences (mus musculus) in qRT-PCR

| Primer | Forward (5’ to 3’) | Reverse (5’ to 3’) |
| --- | --- | --- |
| *Nfe2l1* | TTGGCTCTACCAACCTAGCAG | CCAGCATAGCTTCGTCTAACAG |
| *Psma7* | AACGTCTGTATGGCCTTTGC | GTCACTGGGTCCTCCACTGT |
| *Psmb4* | TTCACTGGCCACTGGTTATG | CGAACGGGCATCTCTGTAGT |
| *Psmb7* | CTGTCTTGGAAGCGGATTTC | GCAACAACCATCCCTTCAGT |
| *Psmc1* | AAGGGGGTCATTCTCTACGG | AAGCTCTGAGCCAACCACTC |
| *Psmc4* | TGGTCATCGGTCAGTTCTTG | CGGTCGATGGTACTCAGGAT |
| *Psmd1* | GGGGCTTTTGAGGAGTCTCT | GCAAATCTGCATTTTCCACA |
| *Psmd12* | TCACAGACCTGCCAGTCAAG | AGGTTTTAGTCAGCCGAGCA |
| *Nqo1* | AGGATGGGAGGTACTCGAATC | TGCTAGAGATGACTCGGAAGG |
| *Tnfa* | AGACCCTCACACTCAGATCA | TCTTTGAGATCCATGCCGTTG |
| *Il1b* | GAAATGCCACCTTTTGACAGTG | TGGATGCTCTCATCAGGACAG |
| *Il10* | CTTACTGACTGGCATGAGGATCA | GCAGCTCTAGGAGCATGTGG |
| *Gapdh* | AACTTTGGCATTGTGGAAGG | GGATGCAGGGATGATGTTCT |

**Supplemental Figures**


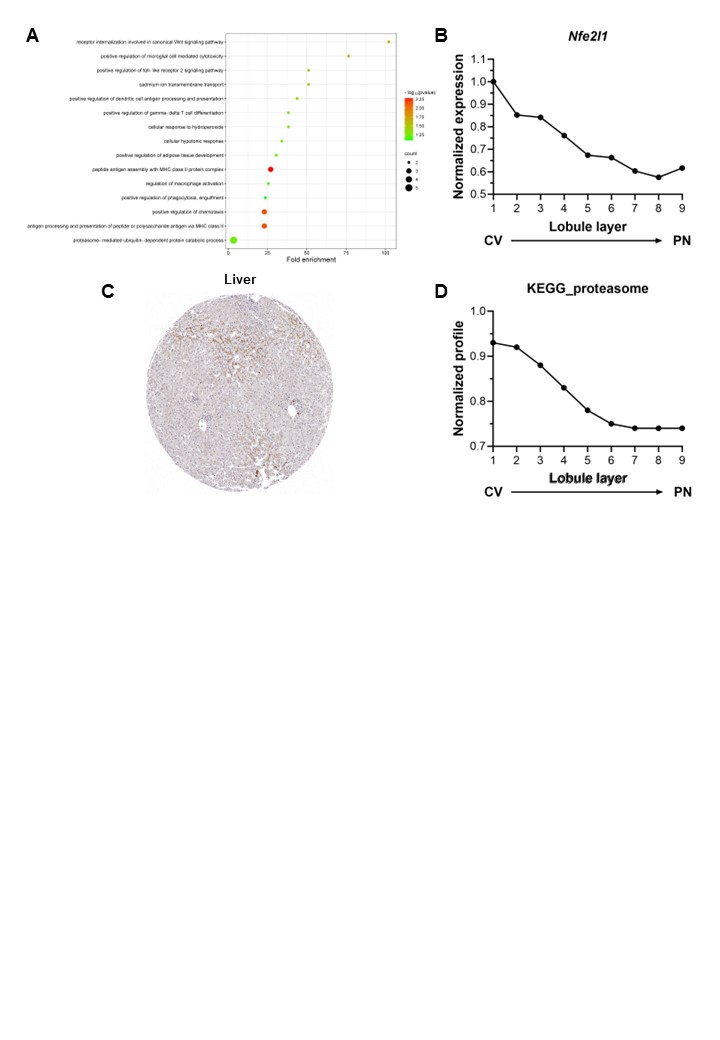


**Figure S1. Zonation profiles of *Nfe2l1* in liver.** (**A**) Bubble diagram presenting KEGG pathway enrichment analysis of MASH patients compared with MAFL patients (GSE105127). (**B**) Zonation profiles of *Nfe2l1* (GSE84498). *x* axis is the scaled distance from the central vein, *y* axis is the max-normalized expression level. (**C**) The immunohistochemical staining of NRF1 in mice livers. (**D**) Zonation profiles of proteasome pathway through KEGG enrichment analysis (GSE84498). CV: central vein; PN: portal node.


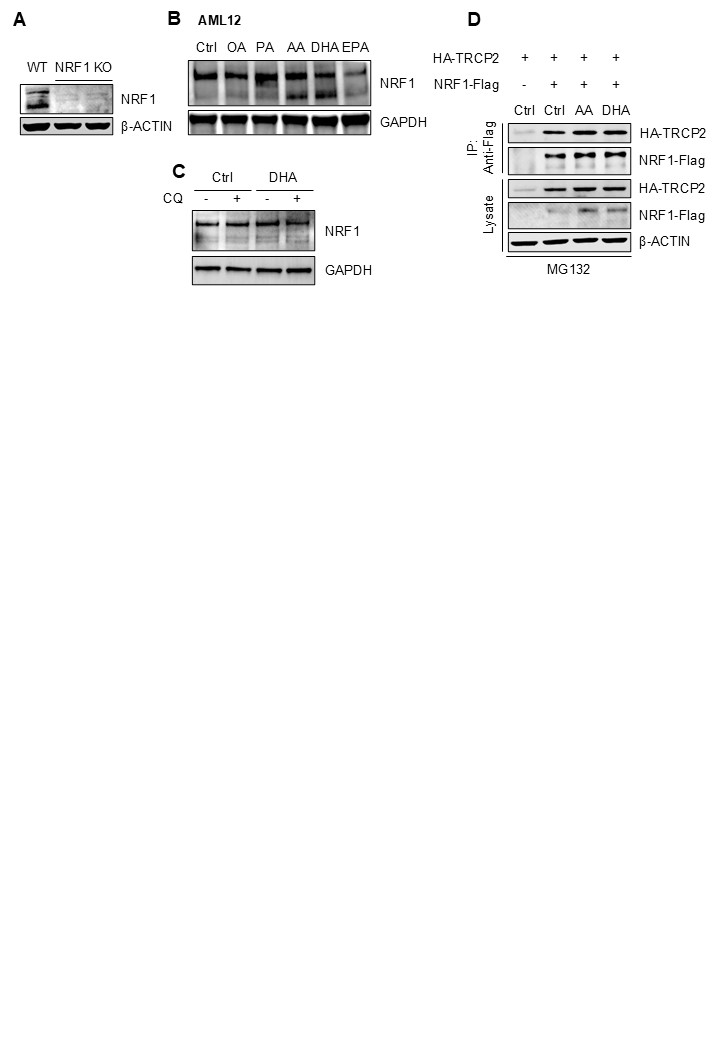


**Figure S2. The regulatory mechanism of NRF1 by DHA.** **(A)** Verification of NRF1-deficient AML12 cell lines by immunoblotting. **(B)** Western blot analysis of NRF1 expression in AML12 cells stimulated with various fatty acids of 0.1 mM for 12 h. **(C)** Western blot analysis of NRF1 expression in HepG2 cells with or without CQ treatment. **(D)** The coimmunoprecipitation assay reflecting interaction between NRF1-Flag and HA-TRCP2 after stimulation with various fatty acids in HepG2 cells.


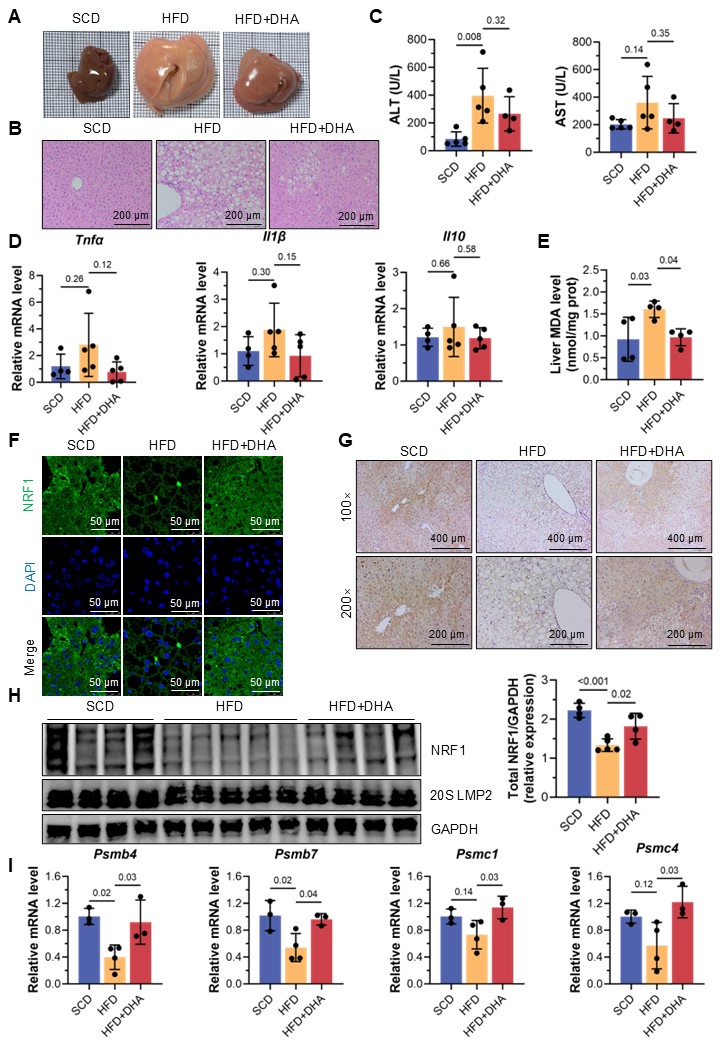


**Figure S3.** **The protective effect of DHA against MASH.** (**A**) Gross observation of liver tissues from mice fed a SCD, a purely HFD or a HFD supplemented with 4% DHA for 20 weeks. (**B**) H&E staining of liver tissues from the abovementioned mice. (**C**) Aminotransferase levels in serum from the abovementioned mice (SCD, *n* = 5; HFD, *n* = 5; HFD+DHA, *n* = 4). (**D**) Gene expression of inflammatory factors (*Tnfα*, *Il1β* and *Il10*) in liver tissues from the abovementioned mice (SCD, *n* = 4; HFD, *n* = 5; HFD+DHA, *n* = 5). (**E**) The MDA level of liver tissues from the abovementioned mice (*n* = 4 for each group). (**F**) Immunofluorescence of NRF1 in liver tissues from the abovementioned mice. (**G**) Immunochemical staining of NRF1 in liver tissues from the abovementioned mice. (**H**) Western blot analysis of NRF1 expression in liver tissues from the abovementioned mice (SCD, *n* = 4; HFD, *n* = 5; HFD+DHA, *n* = 4). (**I**) Gene expression of proteasome subunits (*Psmb4*, *Psmb7*, *Psmc1* and *Psmc4*) in liver tissues from the abovementioned mice (SCD, *n* = 3; HFD, *n* = 4; HFD+DHA, *n* = 3). The data were plotted as the means ± SEMs. One-way ANOVA in C, D, E, H and I was used for the statistical analyses.


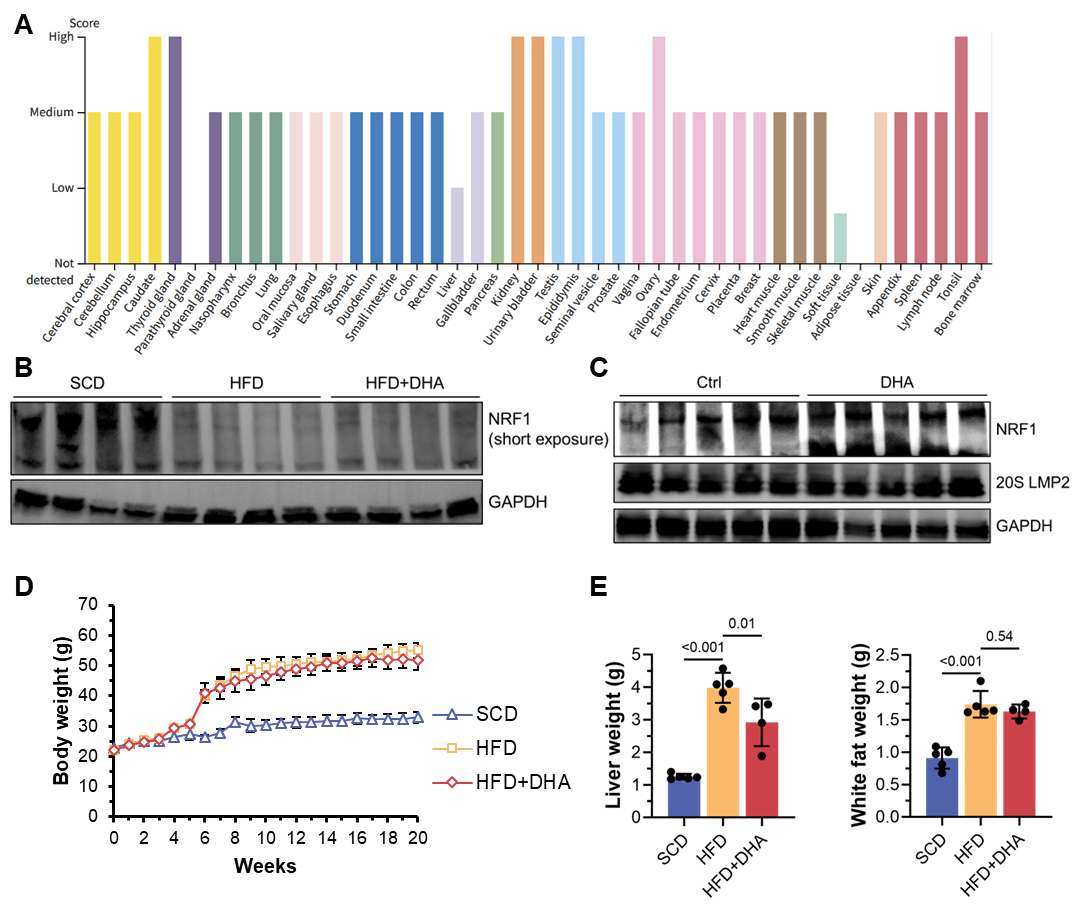


**Figure S4. NRF1 was upregulated by DHA in white adipose tissue.** (A) The expression of NRF1 in various tissues. Data shown were obtained from the Human Protein Atlas database (https://www.proteinatlas.org/). (B) Western blot analysis of NRF1 expression in white adipose tissues from mice fed a SCD, a purely HFD or a HFD supplemented with 4% DHA for 20 weeks (SCD, *n* = 4; HFD, *n* = 4; HFD+DHA, *n* = 4). (C) Western blot analysis of NRF1 expression in SW872 cells stimulated with DHA. (D) Body weight of mice mentioned in the Figure S4B. (E) Liver weight and white fat weight of mice mentioned in the Figure S4E. The data were plotted as the means ± SEMs. One-way ANOVA in E was used for the statistical analyses.


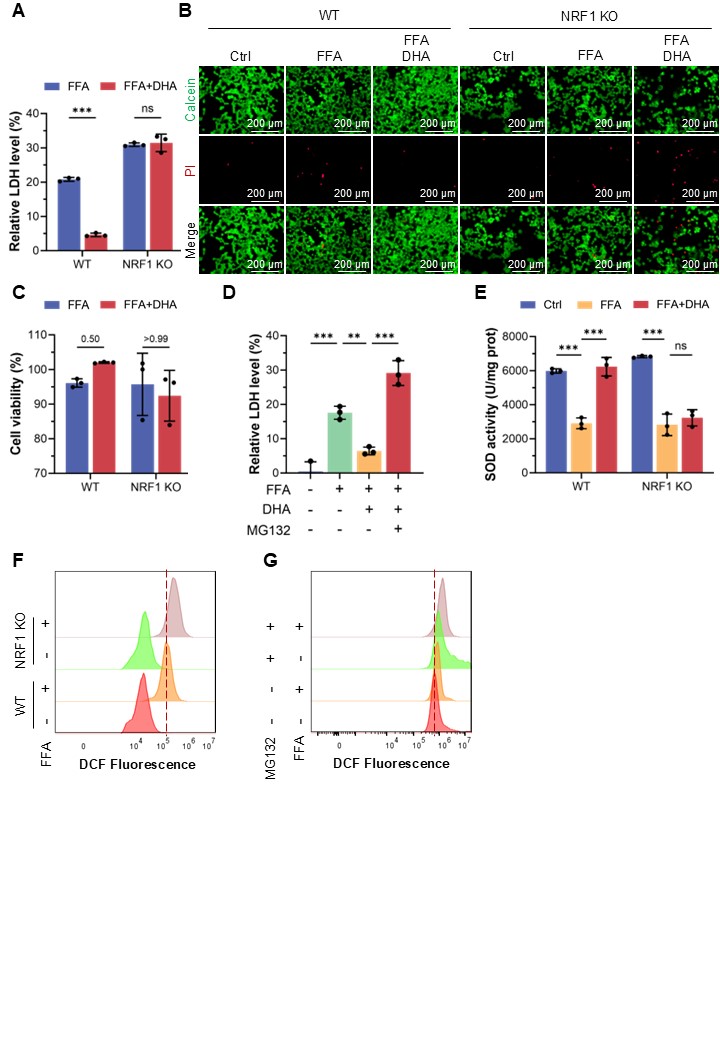


**Figure S5. Absence of NRF1 reversed the protective effect of DHA against liver injury in MASH.** **(A-C)** The LDH level in the supernatant (**A**), the calcein/PI staining (**B**) and cell viability evaluated by CCK-8 assay (**C**) of WT and NRF1-defecient AML12 cells stimulated with FFAs together with or without DHA (*n* = 3 for each group). **(D)** The LDH level in the supernatant of AML12 cells treated with proteasome inhibitor MG132 (10 μM) for 6 h after stimulation of FFA and DHA (*n* = 3 for each group). (**E**) The SOD activity of WT and NRF1-defecient AML12 cells stimulated with FFAs together with or without DHA (*n* = 3 for each group). (**F**) Intracellular ROS level as determined using flow cytometry in WT and NRF1-defecient AML12 cells treated with FFAs. (**G**) Intracellular ROS level as determined using flow cytometry in AML12 cells stimulated with FFAs together with MG132. The data were plotted as the means ± SEMs. Two-way ANOVA in A, C and E and one-way ANOVA in D were used for the statistical analyses. ns: not significant, ** *P* < 0.01, *** *P* < 0.001.


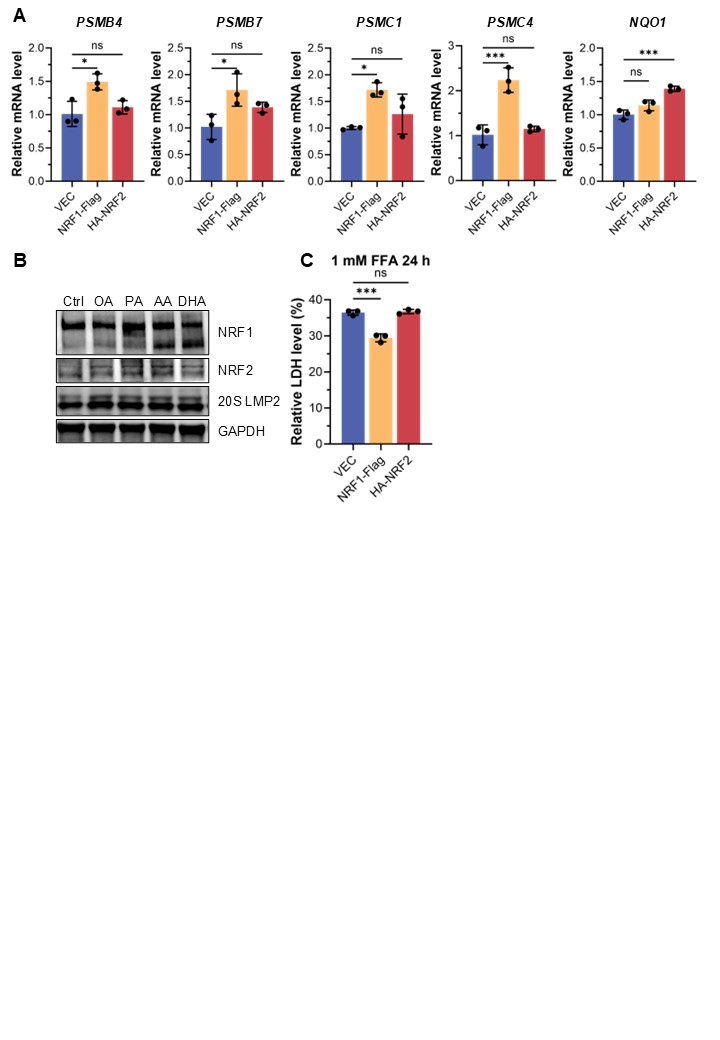


**Figure S6. NRF1 rather than NRF2 was upregulated by DHA to alleviate liver injury in MASH.** (**A**) Transcriptional expression of proteasome subunits (*PSMB4*, *PSMB7*, *PSMC1* and *PSMC4*) and downstream gene of NRF2 (*NQO1*) in HepG2 cells transfected with NRF1-Flag or HA-NRF2 (*n* = 3 for each group). (**B**) Western blot analysis of NRF1 and NRF2 expression in HepG2 cells treated with various fatty acids of 0.1 mM for 12 h. (**C**) The LDH level in the supernatant of HepG2 cells transfected with NRF1-Flag or HA-NRF2 and stimulated with 1 mM FFAs for 24 h (*n* = 3 for each group). The data were plotted as the means ± SEMs. One-way ANOVA in A and C was used for the statistical analyses. ns: non-significance, * *P* < 0.05, *** *P* < 0.001.
